# The Use of Factorial Design and Simplex Optimization to Improve Analytical Performance of In Situ Film Electrodes

**DOI:** 10.3390/s20143921

**Published:** 2020-07-14

**Authors:** Matjaž Finšgar, Klara Jezernik

**Affiliations:** Faculty of Chemistry and Chemical Engineering, University of Maribor, Smetanova ulica 17, 2000 Maribor, Slovenia; klara.jezernik@student.um.si

**Keywords:** factorial design, simplex, optimization, trace heavy metals

## Abstract

This work presents a systematic approach to determining the significance of the individual factors affecting the analytical performance of in-situ film electrode (FE) for the determination of Zn(II), Cd(II), and Pb(II). Analytical parameters were considered simultaneously, where the lowest limit of quantification, the widest linear concentration range, and the highest sensitivity, accuracy, and precision of the method evidenced a better analytical method. Significance was evaluated by means of a fractional factorial (experimental) design using five factors, i.e., the mass concentrations of Bi(III), Sn(II), and Sb(III), to design the in situ FE, the accumulation potential, and the accumulation time. Next, a simplex optimization procedure was employed to determine the optimum conditions for these factors. Such optimization of the in situ FE showed significant improvement in analytical performance compared to the in situ FEs in the initial experiments and compared to pure in situ FEs (bismuth-film, tin-film, and antimony-film electrodes). Moreover, using the optimized in situ FE electrode, a possible interference effect was checked for different species and the applicability of the electrode was demonstrated for a real tap water sample.

## 1. Introduction

Electrochemical measurements of low concentrations of heavy metals are frequently performed using square-wave anodic stripping voltammetry (SWASV) and an in situ film electrode (FE). This is an effective way to determine the traces of various analytes, with short analysis time, and at very low analysis cost. The bismuth-film electrode (BiFE) was introduced by Wang et al. [1,2] as a very promising material that can substitute for Hg electrodes in electroanalysis. Subsequently, an antimony-film electrode was also proposed (SbFE) [3,4], followed by other in situ electrodes. Various modifications of different in situ electrodes have evolved since (some examples are given in references [5,6,7,8,9,10,11,12,13,14,15,16,17,18,19,20,21]).

In the vast majority of cases, in order to achieve optimized conditions, trial-and-error experiments are performed. By doing so, the term optimization is questionable. The so-called optimization is performed by changing one factor while maintaining the others at a certain value to obtain a better analytical response. After getting a better response for a certain factor value (the one that was changed) than that for the initial experiment, the obtained value is then maintained, and the other factors are optimized in the same manner. This procedure is a one-by-one optimization process without the use of a model and usually does not lead to the optimum but only local improvement. Another aspect that needs to be considered is the response. Most frequently a better analytical performance is deemed to have been achieved when a higher stripping peak is obtained. However, the latter would improve the sensitivity (the slope in the case of methods with a linear response) and most likely the limit of detection (LOD) and limit of quantification (LOQ). In the SWASV experiment, improved sensitivity, LOD, and LOQ frequently narrow the linear concentration range [22,23,24,25,26], which is a disadvantage in analytics. Moreover, the question arises as to how the accuracy and precision are affected by such optimization. Optimization can also be performed by scanning different factors in a broad range one-by-one, which is very time consuming and some important aspects can be overlooked. A significantly better approach is to use a proper optimization procedure to reduce the number of experiments and to be able to achieve the optimum conditions. Usually, the mass concentration (*γ*) of the ions to form an in situ FE, accumulation time (*t*_acc_), and accumulation potential (*E*_acc_) are optimized to obtain higher stripping peaks.

This work presents a systematic evaluation of different factors to determine if they have a significant impact on the analytical performance of in situ FEs. In order to determine significance, a factorial design (commonly also referred to as an experimental design) was employed [27,28]. A combination of analytical parameters regarding the linear concentration range, accuracy, precision, LOQ, and sensitivity was considered simultaneously with the analytical performance of the method. It was shown previously that a combination of ions to form an in situ FE can improve certain analytical performance [22,23,24]. On that basis, different ions to form an in situ FE were also used in this work (however, in order to obtain the best analytical performance, a completely different approach was employed herein). As combinations of Bi(III) and Sb(III) were shown previously to be good candidates for in situ FE formation [23], the influence of their *γ* was considered in this study (*γ*_Bi(III)_ and *γ*_Sb(III)_). Additionally, an in situ tin-film electrode (SnFE) was shown previously to have some analytical advantages in electroanalytics [24]; thus, the *γ* of this ion was also considered as one factor to be studied and optimized (*γ*_Sn(II)_). After significance determination and in order to improve the analytical performance of the in situ electrode, a simplex optimization procedure was employed. The latter procedure identified the in situ FE that have significantly better analytical performance compared to the in situ FE that were employed in the initial experiments (before optimization) and pure in situ FE that were formed at the same *γ* as for a combination of ions. Moreover, the influence of different species on a possible interference effect was checked, and the applicability of the optimized in situ electrode for real sample analysis was demonstrated.

This work, therefore, demonstrates that one-by-one optimization cannot achieve such an improvement in analytical performance compared to optimization using a model. Moreover, a weighting factor was suggested for a particular analytical parameter, which can be employed in subsequent studies.

## 2. Experimental

In this work, the in-situ FEs were named by the term XBiYSnZSb, where X, Y, and Z represent the mass concentrations of Bi(III), Sn(II), and Sb(III) in solution to form an in situ FE, respectively. For example, the term 0.60Bi0.80Sn0.30Sb stands for the in situ FE where the 0.1 M acetate buffer solution contained 0.60 mg/L Bi(III), 0.80 mg/L Sn(II), and 0.30 mg/L Sb(III). In cases where the in situ FE was designed in a solution that did not require at least one of the three mentioned ions, the ion that was missing in solution was not included in the designation (e.g., 0.59Bi0.10Sn as no Sb(III) was employed to form the in situ FE). A solution of 0.1 M acetate buffer was used as a supporting electrolyte in all SWASV measurements presented herein.

### 2.1. Solution Preparation

Solutions of standards were prepared using standard stock solutions (1000 mg L^−1^) of Cu(II), Bi(III), Sn(II), Sb(III), As(III), Zn(II), Cd(II), and Pb(II). These solutions were supplied by Merck (Darmstadt, Germany). NaCl, KCl, KNO_3_, C_2_H_4_O_2_, and (NH_4_)_2_Fe(SO_4_)_2_·6H_2_O were supplied by Sigma Aldrich (St. Louis, MO, USA), CaCl_2_ and MgCl_2_ were supplied by Acros Organics (Fair Lawn, NJ, USA), Na_2_SO_4_ was supplied by Honeywell Fluka (Charlotte, NC, USA), and CH_3_COONa·3H_2_O was supplied by Fisher Chemical (Pittsburgh, PA, USA). All dissolutions were performed using ultrapure water (with a resistivity of 18.2 MΩ cm) obtained using the ELGA water purification system (Lane End, UK). All the chemicals were of analytical grade if not stated otherwise.

The real tap water sample was obtained in the laboratory (drinking water). Using this water, a 0.1 M acetate buffer solution was prepared in the same manner as for the model 0.1 buffer solution, except for the usage of tap instead of ultrapure water.

### 2.2. Electrochemical Measurements

All potentials reported in this work refer to the Ag/AgCl(saturated KCl) reference electrode. A platinum wire was used as a counter electrode and a glassy carbon electrode, GCE (a disc with a diameter of 3.0 mm, sealed in Teflon), served as the working electrode (Cat. No. 6.1204.300). Measurements were performed in an electrochemical cell and the volume of the solution for measurements was 20.0 mL (before the addition of the solutions of standard). The cell and the electrodes were supplied by Metrohm (Herisau, Switzerland). The electrochemical measurements were carried out at room temperature using a potentiostat/galvanostat supplied by PalmSens (PalmSens, Houten, the Netherlands), model PalmSens3 EIS. Measurements were controlled with PSTrace 5.6 software (PalmSens).

Before every electrochemical measurement, the GCE surface was polished using 0.05 μm Al_2_O_3_ (Buehler, IL, USA). Then, the electrode was rinsed with ultrapure water, followed by ultrasound cleaning in ultrapure water for 1 min. After the ultrasound cleaning, the electrode was rinsed with ultrapure water and immersed in 15 wt.% HCl for approximately 10 min. A potential of 0.600 V was applied to further perform chemical/electrochemical cleaning. Then, the GCE was thoroughly rinsed with ultrapure water and gently wiped with a paper towel, without touching the active GCE surface. The adequacy of every preparation procedure before the SWASV experiments was tested with a hexacyanoferrate system and cyclic voltammetry, as explained previously [22].

SWASV measurements were performed at different *E*_acc_ and *t*_acc_, as explained below, and using a 50 mV amplitude, a 4 mV potential step, and a frequency of 25 Hz. The equilibration time was always 15 s. During the accumulation and cleaning steps the solution was stirred at approximately 300 rpm, whereas the solution was not stirred during the equilibration and measurement. After the voltammogram measurement, a potential of 0.600 V was applied for 30 s to remove possible residual metals from the accumulation step. All SWASV measurements were performed in 0.1 M acetate buffer solution at pH 4.5 and all solutions were not deoxygenated.

### 2.3. Evaluation of the Performance of the SWASV Pethod

**Figure 1:**
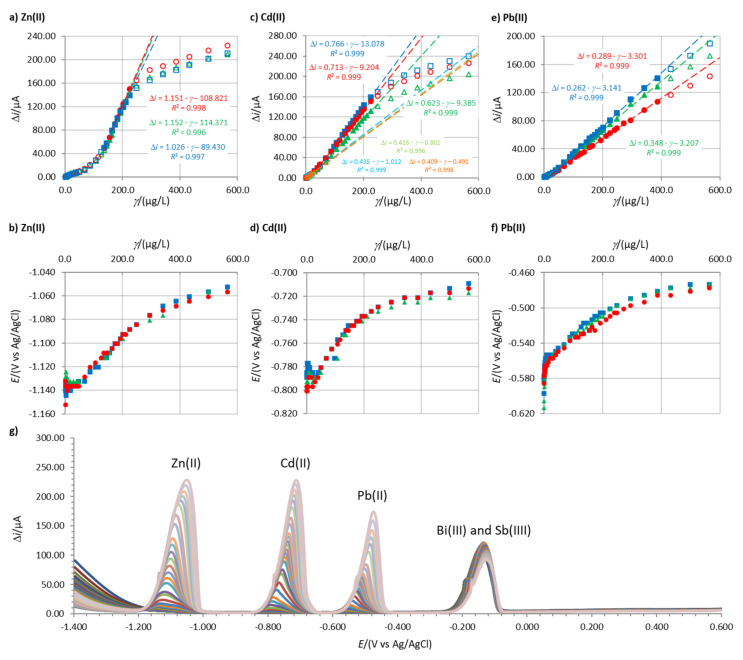
Linear concentration ranges for (**a**) Zn(II), (**c**) Cd(II), and (**e**) Pb(II), and the stripping peak potentials for (**b**) Zn(II), (**d**) Cd(II), and (**f**) Pb(II). The measurements were performed using 1.00Bi0.60Sn0.34Sb in 0.1 M acetate buffer using *t*_acc_ = 100 s and *E*_acc_ = −1.380 V. Figure (**g**) shows the increase in stripping peaks with increasing concentration of the analytes (simultaneously). The full symbols in a,c,e) characterize the linear concentration range, whereas the empty symbols characterize concentrations above and below the linear concentration range.

In general, the desire is always to determine the analytical method with the lowest limit of quantification (LOQ) and the lowest limit of detection (LOD), the highest sensitivity (herein represented by the slope of the calibration curve as the methods showed a linear response with a change in concentration), the highest precision of the method (the lowest relative standard deviation, RSD, for the replicate measurements), the highest accuracy (the best recovery, Re, i.e., the closest to 100.0%), and the widest linear concentration range. Based on the above-mentioned, the performance of the SWASV method was evaluated with these five different analytical parameters, i.e., the LOQ, the width of the linearity range, the slope of the calibration curve, the RSD (obtained from the precision of the method), and the difference of the Re relative to 100.0% (|100.0%−Re|). These analytical parameters are given in Equation (1) to obtain a value for the optimization criterion (OC). As the LOQ was included in this evaluation, the LOD was not considered in the OC calculation as the LOD and LOQ are very similar analytical properties (of course, not the same) and due to that, this property was not considered twice (if the LOQ is at a low concentration, then the LOD is also at a low concentration). All analytical parameters for OC evaluation were measured at least six times (also the method linearity; only three are shown in Figure 1 and in the figures in the Supplementary Materials) and the values were checked for possible outliers using Dixon’s and Grubb’s statistical test at 95% confidence [29]. If detected, they were discarded. In the case of the LOD and LOQ, the modus value was reported among all measurements.

The higher the OC value, the better is the analytical performance of the method. As a wider linear concentration range and the highest sensitivity is desired, the terms describing these analytical parameters are in the numerator of Equation (1). On the other hand, as the lowest RSD, the lowest LOQ, and the Re values closest to 100.0% are desired, these parameters are given in the denominator of Equation (1). Because the Re can be lower or higher than 100.0%, the [100.0% – Re] term is expressed in absolute values.

(1) OCanalyte=(the width of the linear concentration range)analyte·(calibration curve slope)analyteLOQanalyte·RSDanalyte·|100.0%−Re(%)|analyte

In the present case, three analytes were determined simultaneously (Zn(II), Cd(II), and Pb(II)), and therefore every analyte has its own OC. As the electrochemical method was considered for the simultaneous determination of all these analytes, a combined OC should be employed in order to include the analytical properties of all the analytes simultaneously. This combined OC (OC_combined_) was determined as a product of every OC for Zn(II), Cd(II), and Pb(II), as given in Equation (2) and it served as a value to determine which in situ FE performs better.

(2) $${\mathrm{OC}}_{\mathrm{combined}}={\mathrm{OC}}_{\mathrm{Zn}\left(\mathrm{II}\right)}\cdot {\mathrm{OC}}_{\mathrm{Cd}\left(\mathrm{II}\right)}\cdot {\mathrm{OC}}_{\mathrm{Pb}\left(\mathrm{II}\right)}$$

The Re value and RSD (for the precision of the method) for all three analytes were determined at the same concentration, i.e., at the lowest concentration where all three analytes were within their linear concentration ranges simultaneously (if not stated otherwise). It must be pointed out that the Re and RSD were determined by measuring the concentrations of the analytes, where the solution and electrode preparation was carried out every time anew, to test the right precision of the method. Frequently in the literature, RSD values are reported where the same solution is measured in sequence, which stands for the precision of the system (the latter is also reported below), which of course gives different RSD values than that for the precision of the method (the latter are higher or in the best possible case equal, but cannot be lower). Hereinafter, only the numerical values without units are reported for OC_combined_.

Figure 1 shows an example of the linear concentration measurements, the corresponding change in the stripping peak potential, and the increase in stripping peak heights with an increase in the concentration of the analytes. These data for other electrodes that were tested are given in the Supplementary Materials.

## 3. Results and Discussion

### 3.1. Fractional Factorial Design

The influence of different factors on the SWASV analytical performance was investigated by means of a fractional factorial design. Five different factors were considered, i.e., *γ*_Bi(III__)_ (*x*_1_), *γ*_Sn(II)_ (*x*_2_), *γ*_Sb(III)_ (*x*_3_), *E*_acc_ (*x*_4_), and *t*_acc_ (*x*_5_). The fractional factorial design was an extended full two-level factorial design with 3 factors (2^3^). The levels for factors *x*_1_, *x*_2_, and *x*_3_ correspond to a full two-level factorial design with 3 factors, whereas factors *x*_4_ and *x*_5_ are obtained by *x*_1_ · *x*_2_ = *x*_4_ and *x*_1_ · *x*_3_ = *x*_5_, where (+ · + gives +, + · − gives −, and − · − gives +) in order to design the fractional factorial design. The fractional two-level factorial design with five factors is shown in Table 1 (+ stands for the factor when it is at the high level and – stands for the factor when it is at the low level). The most negative *E*_acc_ was set at −1.500 V, as hydrogen evolution is significant at more negative potentials (an example of such hydrogen evolution is given in Figure 1g). The decision level for the concentration of the ions for the formation of the in situ FE was set to 0.50 mg/L, as this is a very common concentration used to form an in situ FE [1,2,3,30,31,32,33,34]. The decision level for the *E*_acc_ was set at −1.350 V, as at potentials more positive than −1.200 V (which is the low level for this factor in Table 1) the accumulation of Zn(II) would be an issue (the Zn(II) stripping peak is at approximately −1.100 V). The decision level for the *t*_acc_ was set to 55 s (60 s is a very common *t*_acc_ [1,2,3,30,31,32,33,34] and therefore this value was included in the high level for *t*_acc_). Table 1 shows the levels for every factor when they are at the high or low level.

Partial method validation by measuring the LOQ (also the LOD, but this was not included in the OC, as mentioned above), linear concentration range (and simultaneously the calibration curve’s slope), RSD, and Re was performed for every in situ FE (the experimental variables are as given in the factorial design in Table 1) and the OC_combined_ values were calculated for each in situ FE. The OC_combined_ values served as a response for the factorial design. The obtained OC_combined_ values, the factor impact, and the critical values are presented in Table 1.

The assessment of whether one factor has a significant impact on the analytical property is determined as $\mathrm{factor}\;\mathrm{impact}=\bar{{\mathrm{OC}}_{{\mathrm{combined}}^{+}}}-\bar{{\mathrm{OC}}_{{\mathrm{combined}}^{-}}}$, where the $\bar{{\mathrm{OC}}_{{\mathrm{combined}}^{+}}}$ and $\bar{{\mathrm{OC}}_{{\mathrm{combined}}^{-}}}$ are the average values of OC_combined_ when the factor is at the high and low level, respectively. The impact of the factor is significant when

$$\left|\mathrm{factor}\;\mathrm{impact}\right|>\mathrm{critical}\;\mathrm{value}=t\left(0.05,\;n_{{\mathrm{OC}}_{\mathrm{combined}}+}+n_{{\mathrm{OC}}_{\mathrm{combined}}-}-2\right)\cdot s_{\mathrm{pooled}}\cdot \sqrt{\frac{1}{n_{{\mathrm{OC}}_{\mathrm{combined}}+}}+\frac{1}{n_{{\mathrm{OC}}_{\mathrm{combined}}-}}},$$

where $s_{\mathrm{pooled}}=\sqrt{\frac{\left(n_{{\mathrm{OC}}_{\mathrm{combined}}+}-1\right)\cdot s_{{\mathrm{OC}}_{\mathrm{combined}}+}^{2}+\left(n_{{\mathrm{OC}}_{\mathrm{combined}}-}-1\right)\cdot s_{{\mathrm{OC}}_{\mathrm{combined}}-}^{2}}{n_{{\mathrm{OC}}_{\mathrm{combined}}-}+n_{{\mathrm{OC}}_{\mathrm{combined}}-}-2}}$. The term *s*_pooled_ stands for the pooled standard deviation. The Student’s *t*-value at 95% confidence is $t\left(0.05,\;\left[n_{{\mathrm{OC}}_{\mathrm{combined}}+}+n_{{\mathrm{OC}}_{\mathrm{combined}}-}-2\right]\right)$ and is, in the present case, determined with 6 degrees of freedom (as 4 factors are at the high level and 4 factors are at the low level). The term $s_{{\mathrm{OC}}_{\mathrm{combined}}+}^{2}$ represents the variance of the OC_combined_ values when the factor is at the high level (for example, in the case of the *γ*_Bi(III)_ factor these OC_combined_ values are 0.002056, 0.000142, 0.000056, and 0.000243). On the other hand, $s_{{\mathrm{OC}}_{\mathrm{combined}}-}^{2}$ is the variance of the OC_combined_ values when the factor is at the low level. The terms $n_{{\mathrm{OC}}_{\mathrm{combined}}+}$ and $n_{{\mathrm{OC}}_{\mathrm{combined}}-}$ are the numbers of cases when the factor is either at the high or low level, respectively (in this case, both are 4) [29].

As given in Table 1, no factor alone (*γ*_Bi(III),_ *γ*_Sn(II),_ *γ*_Sb(III),_ *E*_acc_, and *t*_acc_) has a significant influence on the OC_combined_ value, i.e., on the simultaneous combined analytical performance of all three analytes. Based on that, we can conclude that all these factors have an influence simultaneously and no single factor prevails. Therefore, optimizing only one factor (e.g., the optimization of *t*_acc_ is usually reported in the literature) but holding the other factors at a certain value would not lead to optimized conditions, but only to a local improvement. On the other hand, optimizing the system to obtain the global optimum calls for the optimization of all five factors simultaneously. In this study, the simplex optimization procedure was employed.

For comparison, a significance impact evaluation of a single factor on one analytical parameter was performed and the results are given in Table 2. In these cases, the response in the fractional factorial design was a product or sum of the analytical parameters of all three analytes simultaneously instead of the OC_combined_ (everything else is the same as in Table 1). This evaluation is given only for comparison since the main purpose of this study is to improve the five main analytical parameters simultaneously. Table 2 shows that only *t*_acc_ has a significant impact on the LOQ if the response is taken as a product of the individual LOQs for the three analytes. On the other hand, if the response is taken as the sum of the analytes’ individual analytical parameters, only the *t*_acc_ has a significant impact on the width of the linear concentration range (linearity in Table 2), LOQ, sensitivity (slope in Table 2), and precision of the methods (the RSDs in Table 2). However, as will be shown below, it would be misleading if only *t*_acc_ were optimized as the other four factors changed significantly at the end of the simplex optimization.

### 3.2. Simplex Optimization

In the simplex optimization procedure, the OC_combined_ value was used as the optimization criterion. A higher OC_combined_ value represents an improved value. The same factors were considered as in the factorial design given above. As five factors were optimized, six initial experiments were needed to start the optimization (simplex optimization requires one experiment more than the number of factors). As the OC_combined_ values were already determined for eight experiments, six of the experiments with the highest OC_combined_ values were employed in the simplex optimization. Because two more experiments were performed in the factorial design than the required number of experiments for the simplex optimization, two experiments with the lowest OC_combined_ values (the experiments marked with numbers 5 and 6 in Table 1 and Table 2) were discarded from the optimization procedure.

In using the factors from each experiment, in order to determine each subsequent experiment in the simplex optimization, a reflected point (designated with the letter *B*_i_, representing the best reflected point – the best predicted experiment to perform that would give the highest OC_combined_ value) was calculated. The *B*_i_ point was determined by the reflection of the worst point *W*_i_ (the experiment with the lowest OC_combined_ value) through the centroid (CEN) point, which is calculated from all points except *W*_i_. The subscript i represents the number of simplex reflections. The initial simplex procedure was carried out with an α value of 1, i.e., the *B*_i_ value was calculated as *B*_i_ = (1+α)·CEN – α·*W*_i_. When performing the final four simplex reflections, the α value was reduced from 1 to 0.25 as the simplex approached the optimum.

During the optimization, the boundary conditions were established because in some cases the factor values for the next experiment were determined at values that are impossible or can cause issues. The boundary condition was set to −1.500 V for *E*_acc_ if the calculated value predicted more negative *E*_acc_ than −1.500 V, since the evolution of hydrogen becomes significant at more negative potentials than −1.500 V. The latter was applied for *B*_2_ and *B*_5_. In the case of *B*_7_ and *B*_9_, the negative mass concentration of Sb(III) was calculated and thus the boundary condition of 0.00 mg/L was applied.

For experiments *B*_8_, *B*_10_, and *B*_13_, the determined OC_combined_ values were lower than the OC_combined_ values for *W*_8_, *W*_10_, and *W*_13_. In these cases, as required in the simplex procedure, experiments labelled *W*_8_, *W*_10_, and *W*_13_ were not considered as the worst experiments, whereas the experiments with the second worst (lowest) OC_combined_ value were set as *W*_i_ in the simplex procedure (experiments labelled *W*_9_, *W*_11_, and *W*_14_, were determined as new *W*_i_ experiments in these cases). On that basis, new CEN and new *B*_i_ (*B*_9_, *B*_11_, and *B*_14_) were calculated followed by the simplex optimization procedure.

Table 3 shows the sequence of the simplex optimization procedure and the experiments performed. The optimum was reached using the 1.00Bi0.60Sn0.34Sb in situ FE (experiment *B*_12_), where the best analytical parameters for the simultaneous determination of Zn(II), Cd(II), and Pb(II) were determined. Therefore, the in situ FE at the optimum condition was the in situ FE using *E*_acc_ = −1.380 V, *t*_acc_ = 100 s, *γ*_Bi(III)_ = 1.00 mg/L, *γ*_Sn(II)_ = 0.60 mg/L, and *γ*_Sb(III)_ = 0.34 mg/L. The simplex optimization was concluded as the OC_combined_ values for the *B*_13_ and *B*_14_ were significantly lower than for *B*_12_ [35]. Successful optimization of this in situ FE is clearly noted for *B*_12_ as the OC_combined_ value reached 0.111153. For comparison, the best performing in situ FE before the optimization procedure (in the factorial design) had an OC_combined_ value of 0.002056. Therefore, this optimization increased the OC_combined_ value by two orders of magnitude.

As mentioned above, the best in situ FE was 1.00Bi0.60Sn0.34Sb. In the partial method validation for Cd(II) and Pb(II), Re values of 98.3% and 104.5% were obtained, whereas the RSD values (the precision of the method) were 9.5% and 8.7%, respectively. For Zn(II), a lower accuracy (Re = 50.3%) and precision of the method (RSD = 36.1%) were determined. In order to increase these analytical performance features (the accuracy and precision of the method for Zn(II)), a higher weighting factor for |Re-100.0%|_Zn(II)_ and RSD _Zn(II)_ can be added in the OC_combined_ calculation. However, this is beyond the scope of this investigation and could be a subject for future research.

### 3.3. The Linearity, Sensitivity, LOD, LOQ, and Precision of the System for Different In Situ FEs

**Figure 2:**
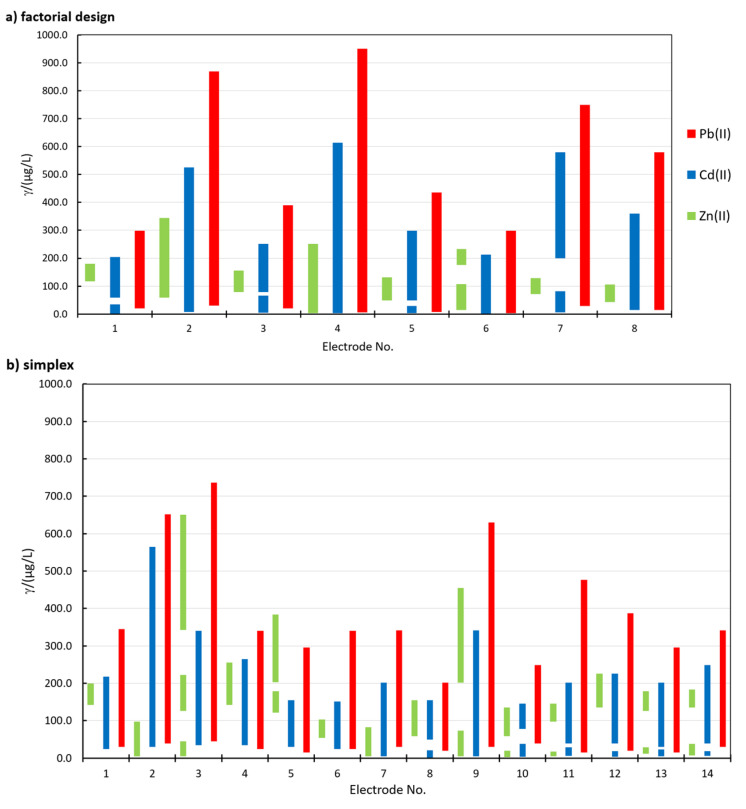
The linear concentration ranges determined for Zn(II), Cd(II), and Pb(II) using different in situ film electrodes (FEs) in (**a**) factorial design and (**b**) the simplex procedure.

Figure 2 shows a representation of the linear concentration ranges for the determination of Zn(II), Cd(II), and Pb(II) using different in situ FEs in the factorial design (Figure 2a) and simplex optimization (Figure 2b). In order to accept a linear concentration range for a certain electrode, two criteria needed to be satisfied, i.e., *R*^2^ > 0.995 and QC < 5.00 % (*R* is the correlation coefficient and *QC* is the quality coefficient [29,36]). The electrode No. for the simplex procedure in Figure 2b represents i, when *B*_i_ was measured.

The width and the range of the linearity for all analytes depend on the in situ FE used. Among the three analytes, in general, the widest linear concentration range was determined for Pb(II), followed by Cd(II), whereas the linear concentration ranges for Zn(II) were the narrowest (for Zn(II), this applies for all in situ FEs apart from 1.15Bi1.22Sn0.57Sb, where the linear concentration range for Zn(II) was wider than that for Cd(II)—electrode No. 5 in Figure 2b). The widest linear concentration range for Pb(II) and Cd(II) was determined in the case of 0.80Bi0.30Sn0.20Sb (electrode No. 4 in Figure 2a), and the widest linear concentration range for Zn(II) was determined for 1.31Bi1.08Sn0.44Sb (electrode No. 8 in Figure 2b).

**Figure 3:**
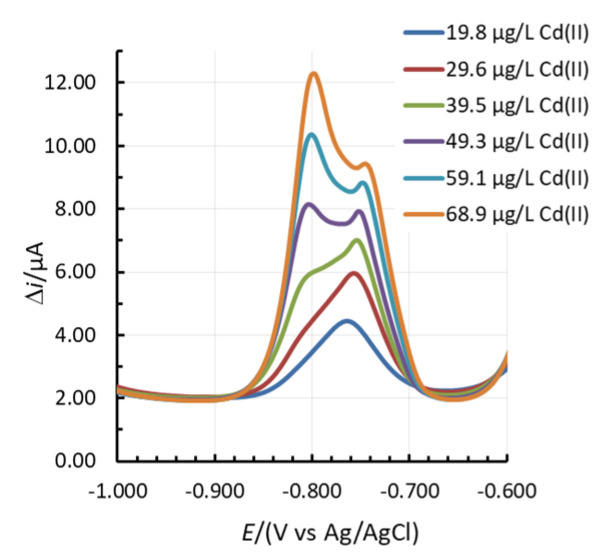
The change in the shape of the Cd(II) stripping peak with an increase in Cd(II) concentration.

**Figure 4:**
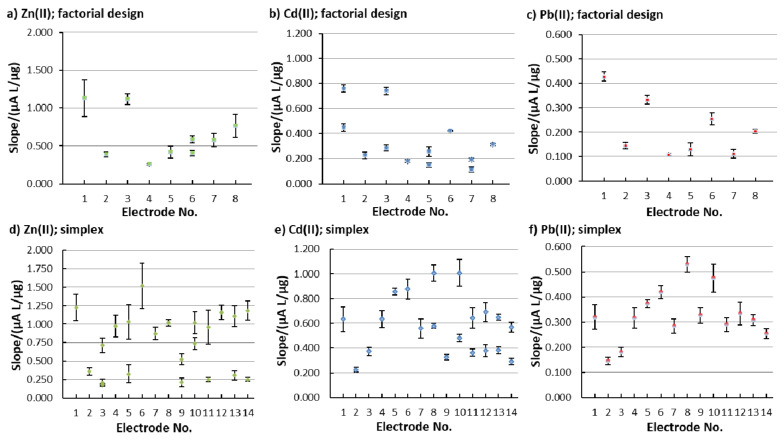
Sensitivity determined as the calibration curve’s slope for the determination of (**a**,**d**) Zn(II), (**b**,**e**) Cd(II), and (**c**,**f**) Pb(II); the slopes were determined in the (**a**–**c**) factorial design and (**d**–**f**) the simplex procedure. The error bars represent the standard deviation of the replicate measurements.

In certain cases, two linear concentration ranges for Zn(II) and Cd(II) developed (one such example for Cd(II) is given in Figure 1c), whereas for 1.31Bi1.08Sn0.44Sb, even three linear concentration ranges were present for Zn(II) (Figure S11 in the Supplementary Materials). In the case of Cd(II), a double current peak for Cd(II) developed. First, the peak at more positive potentials predominates at lower Cd(II) concentrations. With an increase in Cd(II) concentration, the peak at more negative potentials increased, as presented in Figure 3. This shift was previously ascribed to the increased size of the deposited nanoparticles on the surface of the electrode [25,37,38]. Based thereon, also more than one slope value is reported in Figure 4).

For all in situ FEs tested, the three stripping peaks were well separated from each other and from the stripping peaks for the ions that form the in situ FE. Therefore, all electrodes had good selectivity for the determination of these three analytes (one example is given in Figure 1g; more examples are given in the Supplementary Materials).

As mentioned above, sensitivity was evaluated based on the calibration curve’s slope. Figure 4 shows the change in sensitivity (the calibration curve slope values) for the different in situ FEs that were employed in the factorial design (Figure 4a–c) and in the simplex optimization procedure (the electrode No. for the simplex procedure in Figure 4d–f represents i, when *B*_i_ was measured). By comparing the performance of the in situ FEs that were tested in the factorial design with the in situ FEs tested in the simplex optimization, most of the electrodes show a higher sensitivity for the latter.

The LOD is the lowest concentration where the signal can be confidently distinguished from the noise, and the LOQ is the lowest concentration at which quantification is possible. On that basis, the LOD was determined as the concentration at which the signal-to-noise (S/N) ratio was equal or higher than 3.00 (but not exceeding 10.00), whereas the LOQ is the concentration at which the S/N ratio was equal or higher than (but close to) 10 [17,24]. Signal S is the stripping peak’s height and the noise N is the baseline noise (the difference between the highest and the lowest measured point on the baseline). As these values were determined experimentally, the LOQ is not always exactly 3-times (or 3.3-times) higher than the LOD.

Table 4 summarizes the obtained LOD and LOQ values. In general, the lowest LOD and LOQ values were determine for Cd(II). The simplex optimization procedure did not have a significant influence on the LOD and LOQ for these in situ FEs. These values were in the same range as reported previously [22,24,39].

The precision of the system is reported in Table 5 as the RSD obtained for 12 consecutive measurements of the Zn(II), Cd(II), and Pb(II) stripping signals (peak heights) in the same solution. Therefore, this precision is based on the peak height’s determination and not the actual concentration (the latter is commonly determined using the multiple standard addition method). The concentrations at which measurements for the precision of the system were performed correspond to the lowest concentration possible where all three analytes were within their linear concentration range. It was possible to perform the latter for all in situ FEs, apart from 0.30Bi0.30Sn0.60Sb, as the higher limit of the linear concentration range for Zn(II) was at a lower concentration than the lower limit of the second linear concentration range for Cd(II) and close to the higher limit of the first linear concentration range for Cd(II) (see Figure 2b for electrode No. 7). For 0.30Bi0.30Sn0.60Sb, the concentration of the lower limit of the linear concentration range for Zn(II) and the concentration of the lower limit of the linear concentration range for Cd(II) (this was also the concentration of Pb(II)) were tested. The same concentrations as given in Table 5 were also employed to determine the precision of the method and the Re that were incorporated in the OC_combine_ value determination. It would, however, be possible to determine the concentration of all three analytes at 72.2 µg/L using 0.30Bi0.30Sn0.60Sb (as reported in Figure 2b for electrode No. 7), but the concentration determination was performed using the multiple standard addition method, where the linear concentration range of the method needs to be above the value that is being determined. Concentrations were needed to determine the precision of the method and the Re (as mentioned above, they were incorporated into OC_combined_ value determination).

Regarding the vast majority of in situ electrodes, the precision of the system was satisfactory as the RSD values were lower than 20.0%. RSD ≤20.0% is usually the value at which the analytical method is deemed to be precise [40]. However, the 0.64Bi0.83Sn0.01Sb for Zn(II), 0.58Bi0.49Sn0.38Sb for Pb(II), and 1.13Bi0.72Sn0.23Sb for Cd(II) reported RSD values that were above 20.0%, implying the non-precision of the system. On the other hand, it has to be pointed out that the RSD values for the best performing electrode (electrode No. 12 from the simplex optimization) had very low RSD values, i.e., 8.1% for Zn(II), 2.0% for Cd(II), and 7.6% for Pb(II).

It has to be pointed out that the analytical performance of the in situ FE was evaluated by a combination of different analytical parameters for the three analytes (to obtain OC_combined_), and not sensitivity and/or linearity and/or the LOQ alone, as reported above, and the explanation in this section is given only to show how these parameters change during the optimization procedure.

### 3.4. Comparison of the Optimized In Situ FE with the Pure In Situ FEs

The in situ FE design using different ions is reasonable if this combination has better analytical performance compared to the in situ FE design using one ion alone. On that basis, the analytical performance of the optimized 1.00Bi0.60Sn0.34Sb was compared with the pure in situ FE formed using Bi(III) or Sn(II) or Sb(III) alone, by designing 1.94Bi, 1.94Sn, and 1.94Sb in situ FEs. The same partial method validation was performed as above at a mass concentration of 1.94 mg/L for Bi(III) or Sn(II) or Sb(III), which represents the sum of the mass concentrations of the individual ions to design 1.00Bi0.60Sn0.34Sb, i.e., *γ*_Bi(III)_ = 1.00 mg/L, *γ*_Sn(II)_ = 0.60 mg/L, and *γ*_Sb(III)_ = 0.34 mg/L. For pure in situ FEs the same *E*_acc_ = −1.380 V and *t*_acc_ = 100 s were employed as in the case of 1.00Bi0.60Sn0.34Sb. The results are summarized in Table 6. The recovery and precision of the method was tested at the same concentration for all three analytes (for the same reason as mentioned above) for all in situ FEs apart from the 1.94Sb as the upper limit of the linear concentration range for Zn(II) was at a lower concentration than the lower limit of the linear concentration range for Cd(II) and Pb(II).

**Figure 5:**
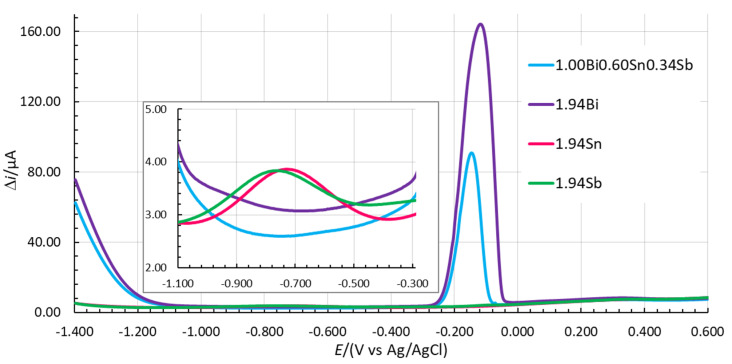
Comparison of voltammograms measured in 0.1 M acetate buffer without analytes using 1.00Bi0.60Sn0.34Sb, 1.94Bi, 1.94Sn, and 1.94Sb in situ FEs (*E*_acc_ = −1.380 V, *t*_acc_ = 100 s).

The LOD and LOQ values for Zn(II), Cd(II), and Pb(II) are similar for 1.00Bi0.60Sn0.34Sb and 1.94Bi. Higher LOD and LOQ values were determined using 1.94Sn, especially for Zn(II). The lowest LOD and LOQ values for Pb(II) were measured for 1.94Sb. On the other hand, using the latter in situ FE, the determination of the LOD and LOQ for Cd(II) was an issue due to the high background contribution in the potential range where the Cd(II) stripping signal occurs (see the insert in Figure 5). When a peak developed that can be clearly distinguished from the baseline, the S/N value was significantly higher than 10 and the reported value can no longer represent the LOQ (and of course not the LOD). The same issue occurred in the case of 1.94Sn and that is why the LOD and LOQ values for these two in situ FEs are not reported in Table 6. On that basis, 1.94Sn and 1.94Sb are less suitable for Cd(II) determination.

The 1.00Bi0.60Sn0.34Sb has the widest linear concentration range and sensitivity for Zn(II). It also has the best accuracy (Re) and precision of the method (RSD) for Cd(II) and Pb(II) among all in situ FEs tested. Using 1.00Bi0.60Sn0.34Sb, the lowest concentrations can be quantified for Cd(II) and Pb(II). The accuracy and precision of the method for the Zn(II) determination are the only parameters that are not satisfactory for this electrode.

As all analytical parameters needed to calculate the OC_combined_ could not be determined with 1.94Sn and 1.94Sb, the OC_combined_ values for these two in situ FEs are not given in Table 6. On the other hand, the OC_combined_ value for 1.94Bi was determined to be 0.009410. BiFE is considered to be one of the benchmarks regarding in situ FEs in the electroanalysis of heavy metal traces [1,8,12,41,42,43,44,45,46,47]. Therefore, the importance of this work is signified with the OC_combined_ value for 1.00Bi0.60Sn0.34Sb, which is at least one order of magnitude better (higher), thus demonstrating the improved design of the in situ FE when using this combination of ions to form an in situ FE compared to the use of only one ion to form a pure in situ FE.

### 3.5. Interference Study

The possible interference effect of different species when present in solution on the Zn(II), Cd(II), and Pb(II) stripping peaks were tested in 0.1 M acetate buffer solution. These species can be present in the water and can potentially increase or decrease the analyte’s stripping peaks and are considered below as potential interferents. The influence of Cu(II), Na(I), Mg(II), As(III), Fe(II), Ca(II), K(I), Cl^−^, SO_4_^2−^, and NO_3_^−^ at three different ratios relative to the mass concentration of the analytes was tested, i.e., 1:1, 1:10, and 1:100 [48]. The concentration of all three analytes was 135.7 µg/L.

The interference effect was determined by measuring the analyte’s stripping peak height with and without the interferent present. The influence of the interferent at a certain analyte:interferent ratio was calculated as [% = 100% (Δ*i*_interferent_–Δ*i*_analite_)/Δ*i*_analite_], where Δ*i*_analite_ is the analyte’s average stripping peak height (3 replicate measurements) and Δ*i*_interferent_ is the analyte’s average stripping peak height in the presence of the interferent (3 replicate measurements). The calculated values are given in Table 7, where positive and negative values represent the increase and decrease in the analyte’s stripping peak height in the presence of the interferent.

**Figure 6:**
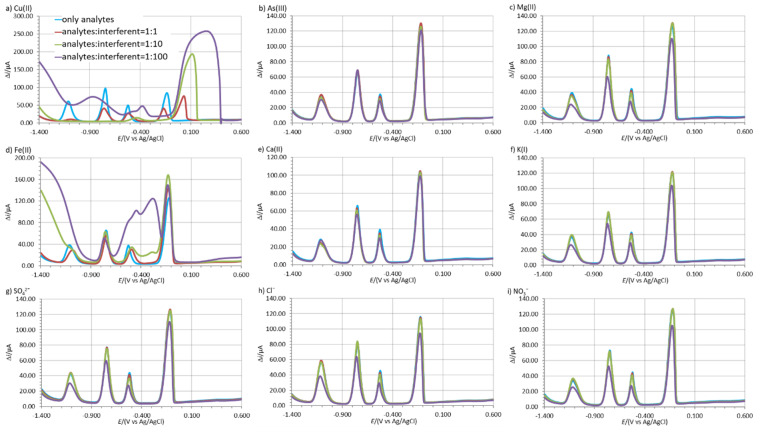
The change in the shape of the voltammograms with and without possible interferents present in solution; (**a**) Cu(II), (**b**) As(III), (**c**) Mg(II), (**d**) Fe(II), (**e**) Ca(II), (**f**) K(I), (**g**) SO_4_^2−^, (**h**) Cl^−^, and (**i**) NO_3_^−^ at a mass concentration ratio of 1:1, 1:10, and 1:100 relative to the analytes. The measurements were performed with 1.00Bi0.60Sn0.34Sb (*E*_acc_ = −1.380 V, *t*_acc_ = 100 s) in 0.1 M acetate buffer containing 135.7 µg/L of all three analytes simultaneously.

Cu(II) and Fe(II) had the greatest influence on the electrochemical determination of Zn(II), Cd(II), and Pb(II). In the case of both of these interferents, the intensity of all three analytes’ stripping peaks decreased significantly even at a concentration ratio of 1:1. All three analytes’ stripping peaks did not developed at a ratio of 1:100, except for the case of *γ*_Cd(II)_:*γ*_Fe(II)_ = 1:100, where the Cd(II) stripping peak developed and its average stripping peak height decreased by 41.7% (Figure 6d and Table 7). The other species tested had a smaller effect on the analytes’ average stripping peak heights (Table 7).

The reason for the above mentioned interference effect can be due to the competitive adsorption of analytes and interferent ions in the case of binary or multicomponent alloy formation during the accumulation procedure [49]. For example, As(III), Sn(II), Sb(III), and Fe(II) were reported before to cause such an interferent effect [3,34,37,43,50,51,52,53,54,55]. On the other hand, the successful alleviation of such an effect can be achieved with the addition of ferrocyanide ions [56,57,58,59]. It must also be pointed out that As(III), Sn(III), and Sb(III) are usually not present in this tap water at such a high concentration and these tests represent a worse-case scenario.

### 3.6. Real Sample Analysis

**Figure 7:**
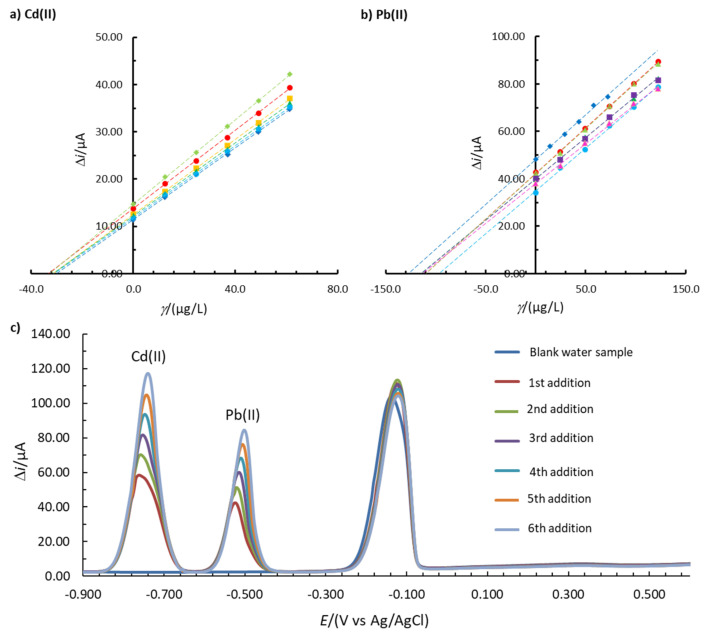
Multiple standard addition method for the analysis of (**a**) Cd(II) and (**b**) Pb(II) using 1.00Bi0.60Sn0.34Sb (*E*_acc_ = −1.380 V, *t*_acc_ = 100 s). Six replicate measurements are shown in Figures a,b. (**c**) One example of the obtained voltammograms is shown in Figure c. The 0.1 M buffer solution was prepared using a real tap water sample.

Real sample analysis was performed with 1.00Bi0.60Sn0.34Sb. As mentioned above, this in situ FE has a high precision of the method and a high accuracy for Cd(II) and Pb(II) determination in the model 0.1 M acetate buffer solution. Based on that, a real sample analysis was performed for these two ions. The tap water sample did not show any stripping peak for Cd(II) or Pb(II) (Figure 7c). The tap water sample was then spiked with Cd(II) and Pb(II) to obtain a final solution of 39.3 µg/L Cd(II) and 135.7 µg/L Pb(II). Figure 7c shows that well-defined Cd(II) and Pb(II) stripping peaks were formed. The analysis was repeated five times and the average Re and RSD values were calculated (no outliers were detected using Dixon’s and Grubb’s tests [29]). To determine the concentration, the multiple standard addition method was employed. The average Re values for Cd(II) and Pb(II) were 83.7% and 88.9%, respectively. The RSD values (demonstrating the precision of the method) were 3.6% and 11.3% for Cd(II) and Pb(II), respectively. As Re was within the interval of 80.0–120.0% and RSD was lower than 20.0% for both analytes [40], it can be concluded that the developed in situ FE is accurate and precise for the determination of Cd(II) in Pb(II) in the real tap water sample.

## 4. Conclusions

A study of the influence of the mass concentration (*γ*) of three ions, Bi(III), Sn(II), and Sb(III) to form an in situ film electrode (FE), the accumulation potential (*E*_acc_), and the accumulation time (*t*_acc_) on the analytical performance of the in situ FE was performed using a fractional two-level factorial design with five factors. The factorial design response was evaluated based on a combination of different analytical parameters, i.e., the limit of quantification (LOQ), sensitivity, linear concentration range, accuracy, and precision. Simultaneously, the lowest LOQ, the highest sensitivity, the highest precision of the method, the highest accuracy, and the widest linear concentration range signify better analytical performance. Based on that, an optimization criterion (OC_analyte_) for the analyte was calculated. As the analysis of three ions was carried out simultaneously (Zn(II), Cd(II), and Pb(II)), the product of OC_Zn(II)_·OC_cd(II)_·OC_Pb(II)_ was considered to be the combined OC, i.e., OC_combined_. A higher OC_combined_ value means better analytical performance. The OC_combined_ value was employed as a response for the factorial design. It was found that none of the factors alone (*γ*_Bi(III)_, *γ*_Sn(II)_, *γ*_Sb(III)_, *E*_acc_, and *t*_acc_) had a significant influence of the final response. It was also found that only *t*_acc_ had a significant influence on the individual analytical parameter, but not all simultaneously. Therefore, the OC_combined_ is dependent on a combination of these factors as it changes when the factors change. On that basis, a simplex optimization procedure was carried out to find the optimized combination of the five factors mentioned above. The OC_combined_ value was employed as the optimization criterion. Such optimization reported the best in situ FE (with the best analytical properties) with the following factors to design an in situ electrode; *γ*_Bi(III)_ = 1.00 mg/L, *γ*_Sn(II)_ = 0.60 mg/L, and *γ*_Sb(III)_ = 0.34 mg/L, *E*_acc_ = −1.380 V, *t*_acc_ = 100 s. This in situ FE was named 1.00Bi0.60Sn0.34Sb. Using this electrode, the OC_combined_ value was significantly higher than that for the benchmark Bi-film electrode.

As five parameters were considered for each analyte (15 together to calculate the OC_combined_), the accuracy and precision of the method was somehow overshadowed and was lower for Zn(II). In order to increase these two analytical performance aspects for Zn(II), a higher weighting factor needs to be taken into account for these two parameters. The latter can be a subject of future research.

It was further demonstrated that 1.00Bi0.60Sn0.34Sb is selective for the determination of Zn(II), Cd(II), and Pb(II). The influence of the presence of different species (Cu(II), Mg(II), As(III), Fe(II), Ca(II), K(I), Cl^−^, SO_4_^2−^, and NO_3_^−^) on the Zn(II), Cd(II), and Pb(II) stripping peak heights was tested to investigate a possible interference effect using 1.00Bi0.60Sn0.34Sb. Among these species, Cu(II) and Fe(II) can be considered to be interferents, whereas the other species had a minor effect or the effect was non-significant. Practical use of 1.00Bi0.60Sn0.34Sb was demonstrated for Cd(II) and Pb(II) analysis in a real tap water sample, where the precision of the method and accuracy was satisfactory for the determination of Cd(II) and Pb(II), i.e., RSD < 20.0% and recovery within an 80.0–120.0% interval.

This work in particular demonstrates that one-by-one optimization (the change of one factor while holding the others at a certain value) does not lead to the optimal values (but only, if that, to local improvement) of the in situ FE with regard to analytical performance. To properly optimize the performance of in situ FEs, the factors to be optimized need to be optimized simultaneously.

## Figures and Tables

**Table 1:** Fractional two-level factorial design with five factors. The color highlights represent the factors at the high and low levels.

Experiment No.	*x*_1_	*x*_2_	*x*_3_	*x*_4_	*x*_5_	*γ*_Bi(III)_ [µg/L]		*γ*_Sn(II)_ [µg/L]	*γ*_Sb(III)_ [µg/L]	*E*_acc_ [V]	*t*_acc_ [s]	OC_combined_
1	+	+	+	+	+	0.80		0.70	0.80	−1.500	90	0.002056
2	+	+	−	+	−	0.60		0.80	0.30	−1.500	30	0.000142
3	+	−	+	−	+	0.70		0.20	0.70	−1.300	80	0.000056
4	+	−	−	−	−	0.80		0.30	0.20	−1.300	20	0.000243
5	−	+	+	−	−	0.20		0.80	0.80	−1.200	40	0.000015
6	−	+	−	−	+	0.20		0.70	0.30	−1.200	70	0.000008
7	−	−	+	+	−	0.30		0.30	0.60	−1.400	30	0.000026
8	−	−	−	+	+	0.20		0.20	0.20	−1.400	60	0.000094
Decision level							0.50	0.50	0.50	−1.350	55	
Factor impact							0.000588	0.000450	0.000417	0.000499	0.000447	
Critical value							0.001172	0.001232	0.001244	0.001213	0.001233	
Significant impact?							No	No	No	No	No	

**Table 2:** The significance of factors on an individual analytical parameter (no or yes is the answer if the factor has a significant impact on the given response). The RSD in % was defined as the precision of the method.

Response	*γ*_Bi(III)_	*γ*_Sn(II)_	*γ*_Sb(III)_	*E*_acc_	*t*_acc_
**response as a combination of the product**					
OC_LOD_ = linearity_Zn(II)_ · linearity_Cd(II)_ · linearity_Pb(II)_	No	No	No	No	No
OC_LOD_ = LOD_Zn(II)_ · LOD_Cd(II)_ · LOD_Pb(II)_	No	No	No	No	No
OC_LOQ_ = LOQ_Zn(II)_ · LOQ_Cd(II)_ · LOQ_Pb(II)_	No	No	No	No	Yes
OC_slope_ = slope_Zn(II)_ · slope_Cd(II)_ · slope_Pb(II)_	No	No	No	No	No
OC_RSD_ = RSD_Zn(II)_ · RSD_Cd(II)_ · RSD_Pb(II)_	No	No	No	No	No
OC_Re_ = |100–Re_Zn(II)_| · |100–Re_Cd(II)_| · |100–Re_Pb(II)_|	No	No	No	No	No
**response as a combination of the sum**					
OC_LOD_ = linearity_Zn(II)_ + linearity_Cd(II)_ + linearity_Pb(II)_	No	No	No	No	Yes
OC_LOD_ = LOD_Zn(II)_ + LOD_Cd(II)_ + LOD_Pb(II)_	No	No	No	No	No
OC_LOQ_ = LOQ_Zn(II)_ + LOQ_Cd(II)_ + LOQ_Pb(II)_	No	No	No	No	Yes
OC_slope_ = slope_Zn(II)_ + slope_Cd(II)_ + slope_Pb(II)_	No	No	No	No	Yes
OC_RSD_ = RSD_Zn(II)_ + RSD_Cd(II)_ + RSD_Pb(II)_	No	No	No	No	Yes
OC_Re_ = |100–Re_Zn(II)_| + |100–Re_Cd(II)_| + |100–Re_Pb(II)_|	No	No	No	No	No

**Table 3:** The simplex optimization procedure with designated *W*_i_ and *B*_i_.

Experiment No.	*γ*_Bi(III)_ [mg/L]	*γ*_Sn(II)_ [mg/L]	*γ*_Sb(III)_ [mg/L]	*E*_acc_ [V]	*t*_acc_ [s]	OC_combined_	
**Factorial Design**							
1	0.80 (3.8 µmol/L)	0.70 (5.9 µmol/L)	0.80 (6.6 µmol/L)	−1.500	90	0.002056	*W*_7_
4	0.80 (3.8 µmol/L)	0.30 (2.5 µmol/L)	0.20 (1.6 µmol/L)	−1.300	20	0.000243	*W*_5_
2	0.60 (2.9 µmol/L)	0.80 (6.7 µmol/L)	0.30 (2.5 µmol/L)	−1.500	30	0.000142	*W*_4_
8	0.20 (1.0 µmol/L)	0.20 (1.7 µmol/L)	0.20 (1.6 µmol/L)	−1.400	60	0.000094	*W*_3_
3	0.70 (3.3 µmol/L)	0.20 (1.7 µmol/L)	0.70 (5.7 µmol/L)	−1.300	80	0.000056	*W*_2_
7	0.30 (1.4 µmol/L)	0.30 (2.5 µmol/L)	0.60 (4.9 µmol/L)	−1.400	30	0.000026	*W*_1_
5	0.20 (1.0 µmol/L)	0.80 (6.7 µmol/L)	0.80 (6.6 µmol/L)	−1.200	40	0.000015	
6	0.20 (1.0 µmol/L)	0.70 (5.9 µmol/L)	0.30 (2.5 µmol/L)	−1.200	70	0.000008	
**Simplex**							
*B*_1_	0.94 (4.5 µmol/L)	0.58 (4.9 µmol/L)	0.28 (2.3 µmol/L)	−1.400	82	0.005867	*W*_13_
*B*_2_	0.64 (3.1 µmol/L)	0.83 (7.0 µmol/L)	0.01 (0.1 µmol/L)	−1.540 *	33	0.003432	*W*_8_, *W*_10_, *W*_12_
*B*_3_	1.31 (6.3 µmol/L)	1.08 (9.1 µmol/L)	0.44 (3.6 µmol/L)	−1.480	42	0.001847	*W*_6_
*B*_4_	1.19 (5.7 µmol/L)	0.60 (5.1 µmol/L)	0.39 (3.2 µmol/L)	−1.372	77	0.018896	
*B*_5_	1.15 (5.5 µmol/L)	1.22 (10.3 µmol/L)	0.57 (4.7 µmol/L)	−1.601 *	109	0.005121	*W*_9_
*B*_6_	0.58 (2.8 µmol/L)	0.49 (4.1 µmol/L)	0.38 (3.1 µmol/L)	−1.429	114	0.006527	*W*_14_
*B*_7_	1.00 (4.8 µmol/L)	0.79 (6.7 µmol/L)	−0.15 ** (−1.2 µmol/L)	−1.380	76	0.013090	
*B*_8_	1.31 (6.3 µmol/L)	0.64 (5.7 µmol/L)	0.64 (5.3 µmol/L)	−1.332	151	0.001571	
*B*_9_	0.59 (2.8 µmol/L)	0.10 (0.8 µmol/L)	−0.14 ** (−1.1 µmol/L)	−1.332	43	0.003460	*W*_11_
*B*_10_	1.08 (5.2 µmol/L)	0.19 (1.6 µmol/L)	0.41 (3.4 µmol/L)	−1.265	124	0.000383	
*B*_11_	0.94 (4.5 µmol/L)	0.80 (6.7 µmol/L)	0.30 (2.5 µmol/L)	−1.437	85	0.046550	
*B*_12_	1.00 (4.8 µmol/L)	0.60 (5.1 µmol/L)	0.34 (2.8 µmol/L)	−1.380	100	0.111153	
*B*_13_	0.94 (4.5 µmol/L)	0.67 (5.6 µmol/L)	0.28 (2.3 µmol/L)	−1.399	93	0.001522	
*B*_14_	1.13 (5.4 µmol/L)	0.72 (6.1 µmol/L)	0.23 (1.9 µmol/L)	−1.385	76	0.001275	

* A boundary condition of −1.500 V was applied. ** A boundary condition of 0.00 mg/L was applied.

**Table 4:** The limit of detection (LOD) and limit of quantification (LOQ) values determined during the factorial design and simplex optimization. S/N ≥ 3.00 and ≥10.00 were taken into consideration for LOD and LOQ determination, respectively.

Electrode No.	Electrode Designation	Zn(II)		Cd(II)		Pb(II)	
		LOD [µg/L]	LOQ [µg/L]	LOD [µg/L]	LOQ [µg/L]	LOD [µg/L]	LOQ [µg/L]
**Factorial Design**							
1	0.80Bi0.70Sn0.80Sb	2.5	3.5	0.5	1.5	1.0	3.0
2	0.60Bi0.80Sn0.30Sb	1.5	3.5	1.0	3.0	1.5	4.5
3	0.70Bi0.20Sn0.70Sb	2.5	3.0	0.5	1.5	1.0	3.0
4	0.80Bi0.30Sn0.20Sb	2.0	3.0	1.0	3.0	1.0	4.0
5	0.20Bi0.80Sn0.80Sb	3.5	5.5	2.0	3.5	2.0	4.5
6	0.20Bi0.70Sn0.30Sb	3.0	3.5	1.0	1.5	1.0	2.5
7	0.30Bi0.30Sn0.60Sb	2.0	3.0	2.5	5.5	3.0	6.5
8	0.20Bi0.20Sn0.20Sb	1.0	2.5	1.5	3.0	1.0	2.5
**Simplex**							
1	0.94Bi0.58Sn0.28Sb	2.5	3.7	0.7	1.7	1.2	3.2
2	0.64Bi0.83Sn0.01Sb	2.7	4.5	1.7	4.0	1.5	4.0
3	1.31Bi1.08Sn0.44Sb	4.0	5.0	1.0	2.7	1.5	4.7
4	1.19Bi0.60Sn0.39Sb	2.5	3.5	0.5	1.2	1.0	2.5
5	1.15Bi1.22Sn0.57Sb	3.2	4.2	0.5	1.2	1.0	2.5
6	0.58Bi0.49Sn0.38Sb	1.7	2.5	0.5	1.2	0.5	1.5
7	1.00Bi0.79Sn	1.5	2.5	0.7	1.5	0.7	2.7
8	1.31Bi0.64Sn0.64Sb	2.5	3.0	0.5	1.0	1.0	2.2
9	0.59Bi0.10Sn	1.7	3.2	0.7	2.0	1.2	3.5
10	1.08Bi0.19Sn0.41Sb	3.2	3.7	0.5	1.0	1.0	2.5
11	0.94Bi0.80Sn0.30Sb	2.5	3.5	0.7	1.7	1.2	2.7
12	1.00Bi0.60Sn0.34Sb	3.0	4.0	0.5	1.2	1.2	2.7
13	0.94Bi0.67Sn0.28Sb	2.5	3.2	0.5	1.5	1.0	2.7
14	1.13Bi0.72Sn0.23Sb	2.0	2.7	0.7	1.7	1.5	3.5

**Table 5:** RSD values, representing the precision of the system obtained during the factorial design and simplex optimization procedure.

Electrode No.	Electrode Designation		Zn(II)	Cd(II)	Pb(II)
		*γ* [µg/L]	RSD [%]	RSD [%]	RSD [%]
**Factorial Design**					
1	0.80Bi0.70Sn0.80Sb	117.5	8.3	1.8	4.0
2	0.60Bi0.80Sn0.30Sb	59.1	14.8	3.7	9.6
3	0.70Bi0.20Sn0.70Sb	78.7	4.6	7.2	7.2
4	0.80Bi0.30Sn0.20Sb	10.0	17.6	2.9	4.1
5	0.20Bi0.80Sn0.80Sb	49.3	6.6	2.1	5.3
6	0.20Bi0.70Sn0.30Sb	14.8	11.8	8.0	12.4
7	0.30Bi0.30Sn0.60Sb		5.0 (72.2 µg/L)	5.3 (199.5 µg/L)	2.1 (199.5 µg/L)
8	0.20Bi0.20Sn0.20Sb	43.4	2.3	2.3	1.4
**Simplex**					
1	0.94Bi0.58Sn0.28Sb	141.6	1.5	7.2	4.3
2	0.64Bi0.83Sn0.01Sb	39.3	26.8	18.0	10.4
3	1.31Bi1.08Sn0.44Sb	126.2	13.0	5.9	3.9
4	1.19Bi0.60Sn0.39Sb	141.6	6.7	1.0	6.0
5	1.15Bi1.22Sn0.57Sb	121.4	9.5	2.0	7.4
6	0.58Bi0.49Sn0.38Sb	54.2	13.2	2.8	20.7
7	1.00Bi0.79Sn	29.6	9.3	13.8	12.0
8	1.31Bi0.64Sn0.64Sb	58.8	5.3	8.6	16.3
9	0.59Bi0.10Sn	29.6	2.2	2.0	17.3
10	1.08Bi0.19Sn0.41Sb	78.1	3.2	7.9	17.3
11	0.94Bi0.80Sn0.30Sb	97.4	3.8	2.4	6.8
12	1.00Bi0.60Sn0.34Sb	135.7	8.1	2.0	7.6
13	0.94Bi0.67Sn0.28Sb	126.2	2.8	6.7	6.2
14	1.13Bi0.72Sn0.23Sb	135.7	13.4	20.1	2.2

**Table 6:** A comparison of the analytical performance of 1.00Bi0.60Sn0.34Sb with 1.94Bi, 1.94Sn, and 1.94Sb. The Re and RSD values were determined at the concentration given in brackets.

In Situ FE	1.00Bi0.60Sn0.34Sb	1.94Bi	1.94Sn	1.94Sb
**Zn(II)**				
Linearity [µg/L]	135.7–225.6	5.0–82.9	**	10.0–64.9
Slope [µA·L/µg]	1.157	0.830	**	0.932
LOD [µg/L]	3.0	3.2	39.2	1.0
LOQ [µg/L]	4.0	3.7	49.0	1.5
Re [%]	50.3	105.6	**	28.1
	(135.7 µg/L)	(29.6 µg/L)		(10.0 µg/L)
RSD [%]	36.1	19.7	**	43.1
**Cd(II)**				
Linearity [µg/L]	4.0–19.8	10.0–154.8	19.9–225.6	16.9–159.5
	39.3–225.6			
Slope [µA·L/µg]	0.379	0.657	0.323	0.479
	0.691			
LOD [µg/L]	0.5	0.5	*	*
LOQ [µg/L]	1.2	1.0	*	*
Re [%]	98.3	89.7	80.2	60.5
	(135.7 µg/L)	(29.6 µg/L)	(63.2 µg/L)	(55.3 µg/L)
RSD [%]	9.5	10.3	33.4	17.1
**Pb(II)**				
Linearity [µg/L]	19.8–386.8	29.6–341.3	34.4–341.3	55.3–1130.9
Slope [µA·L/µg]	0.333	0.326	0.250	0.060
LOD [µg/L]	1.2	1.2	7.4	7.4
LOQ [µg/L]	2.7	3.7	10.3	12.4
Re [%]	104.5	67.1	52.6	68.8
	(135.7 µg/L)	(29.6 µg/L)	(63.2 µg/L)	(55.3 µg/L)
RSD [%]	8.7	12.6	41.2	31.6
OC_combined_	0.111153	0.009410	***	***

* The high background contribution prevented accurate LOD and LOQ determination. ** The analytical performance is not reported due to the irreproducible linearity range determination for the replicate measurements. *** The OC_combined_ was not determined since it was not possible to determine all of the parameters needed for the calculations.

**Table 7:** The influence of different species as possible interferents on the determination of the analytes’ stripping peaks using 1.00Bi0.60Sn0.34Sb (*E*_acc_ = −1.380 V, *t*_acc_ = 100 s) in 0.1 M acetate buffer containing 135.7 µg/L of all analytes simultaneously. This influence was evaluated with the calculation of $\mathrm{the}\;\mathrm{change}\;\%=\left(\frac{\Delta i_{\mathrm{interferent}}-\Delta i_{\mathrm{analyte}}}{\Delta i_{\mathrm{analyte}}}\right)\cdot 100\%$.

Possible Interferent	Mass Concentration Ratio Zn(II):Interferent			Mass Concentration Ratio Cd(II):Interferent			Mass Concentration Ratio Pb(II):Interferent		
	1:1	1:10	1:100	1:1	1:10	1:100	1:1	1:10	1:100
**Cu(II)**	−90.9	−99.6	*	−58.4	−99.3	*	−49.5	−79.3	*
**Mg(II)**	−3.5	−9.0	−42.1	−1.7	−4.2	−32.7	−4.4	−9.7	−40.3
**As(III)**	3.4	−6.9	−14.5	5.5	2.5	5.5	−7.4	−16.5	−21.0
**Fe(II)**	−28.0	−91.4	*	−14.6	−10.5	−41.7	−22.3	−36.4	*
**Ca(II)**	−8.8	−14.7	−7.3	−2.3	−6.6	−17.5	−5.6	−13.7	−26.8
**K(I)**	10.0	12.3	−28.6	3.6	4.6	−19.0	−3.5	−7.9	−31.8
**Cl^−^**	18.0	16.9	−21.8	2.5	5.1	−18.8	−6.5	−12.0	−36.7
**SO_4_^2−^**	7.1	9.6	−30.3	−0.1	0.1	−22.8	−8.9	−15.1	−38.4
**NO_3_^−^**	10.3	11.4	−23.5	−0.4	−1.2	−27.0	−3.7	−10.7	−39.8

* A clear analyte stripping peak did not develop.

## Supplementary Materials

The following are available online at https://www.mdpi.com/1424-8220/20/14/3921/s1, **Figure S1**: Linear concentration ranges for (a) Zn(II), (c) Cd(II), and (e) Pb(II), and the stripping peak potentials for (b) Zn(II), (d) Cd(II), and (f) Pb(II). The measurements were performed using 0.80Bi0.70Sn0.80Sb in 0.1 M acetate buffer. Figure (g) shows the increase in stripping peaks with increasing concentration of the analytes (simultaneously). The full symbols in Figure (a,c,e) characterize the linear concentration range, whereas the empty symbols characterize concentrations above and below the linear concentration range. **Figure S2**: Linear concentration ranges for (a) Zn(II), (c) Cd(II), and (e) Pb(II), and the stripping peak potentials for (b) Zn(II), (d) Cd(II), and (f) Pb(II). The measurements were performed using 0.60Bi0.80Sn0.30Sb in 0.1 M acetate buffer. Figure (g) shows the increase in stripping peaks with increasing concentration of the analytes (simultaneously). The full symbols in Figure (a,c,e) characterize the linear concentration range, whereas the empty symbols characterize concentrations above and below the linear concentration range. **Figure S3**: Linear concentration ranges for (a) Zn(II), (c) Cd(II), and (e) Pb(II), and the stripping peak potentials for (b) Zn(II), (d) Cd(II), and (f) Pb(II). The measurements were performed using 0.70Bi0.20Sn0.70Sb in 0.1 M acetate buffer. Figure (g) shows the increase in stripping peaks with increasing concentration of the analytes (simultaneously). The full symbols in Figure (a,c,e) characterize the linear concentration range, whereas the empty symbols characterize concentrations above and below the linear concentration range. **Figure S4**: Linear concentration ranges for (a) Zn(II), (c) Cd(II), and (e) Pb(II), and the stripping peak potentials for (b) Zn(II), (d) Cd(II), and (f) Pb(II). The measurements were performed using 0.80Bi0.30Sn0.20Sb in 0.1 M acetate buffer. Figure (g) shows the increase in stripping peaks with increasing concentration of the analytes (simultaneously). The full symbols in Figure (a,c,e) characterize the linear concentration range, whereas the empty symbols characterize concentrations above and below the linear concentration range. **Figure S5**: Linear concentration ranges for (a) Zn(II), (c) Cd(II), and (e) Pb(II), and the stripping peak potentials for (b) Zn(II), (d) Cd(II), and (f) Pb(II). The measurements were performed using 0.20Bi0.80Sn0.80Sb in 0.1 M acetate buffer. Figure g) shows the increase in stripping peaks with increasing concentration of the analytes (simultaneously). The full symbols in Figure (a,c,e) characterize the linear concentration range, whereas the empty symbols characterize concentrations above and below the linear concentration range. **Figure S6**: Linear concentration ranges for (a) Zn(II), (c) Cd(II), and (e) Pb(II), and the stripping peak potentials for (b) Zn(II), (d) Cd(II), and (f) Pb(II). The measurements were performed using 0.20Bi0.70Sn0.30Sb in 0.1 M acetate buffer. Figure (g) shows the increase in stripping peaks with increasing concentration of the analytes (simultaneously). The full symbols in Figure (a,c,e) characterize the linear concentration range, whereas the empty symbols characterize concentrations above and below the linear concentration range. **Figure S7**: Linear concentration ranges for (a) Zn(II), (c) Cd(II), and (e) Pb(II), and the stripping peak potentials for (b) Zn(II), (d) Cd(II), and (f) Pb(II). The measurements were performed using 0.30Bi0.30Sn0.60Sb in 0.1 M acetate buffer. Figure (g) shows the increase in stripping peaks with increasing concentration of the analytes (simultaneously). The full symbols in Figure (a,c,e) characterize the linear concentration range, whereas the empty symbols characterize concentrations above and below the linear concentration range. **Figure S8**: Linear concentration ranges for (a) Zn(II), (c) Cd(II), and (e) Pb(II), and the stripping peak potentials for (b) Zn(II), (d) Cd(II), and (f) Pb(II). The measurements were performed using 0.20Bi0.20Sn0.20Sb in 0.1 M acetate buffer. Figure (g) shows the increase in stripping peaks with increasing concentration of the analytes (simultaneously). The full symbols in Figure (a,c,e) characterize the linear concentration range, whereas the empty symbols characterize concentrations above and below the linear concentration range. **Figure S9**: Linear concentration ranges for (a) Zn(II), (c) Cd(II), and (e) Pb(II), and the stripping peak potentials for (b) Zn(II), (d) Cd(II), and (f) Pb(II). The measurements were performed using 0.94Bi0.58Sn0.28Sb in 0.1 M acetate buffer. Figure (g) shows the increase in stripping peaks with increasing concentration of the analytes (simultaneously). The full symbols in Figure (a,c,e) characterize the linear concentration range, whereas the empty symbols characterize concentrations above and below the linear concentration range. **Figure S10**: Linear concentration ranges for (a) Zn(II), (c) Cd(II), and (e) Pb(II), and the stripping peak potentials for (b) Zn(II), (d) Cd(II), and (f) Pb(II). The measurements were performed using 0.64Bi0.83Sn0.01Sb in 0.1 M acetate buffer. Figure (g) shows the increase in stripping peaks with increasing concentration of the analytes (simultaneously). The full symbols in Figure (a,c,e) characterize the linear concentration range, whereas the empty symbols characterize concentrations above and below the linear concentration range. **Figure S11**: Linear concentration ranges for (a) Zn(II), (c) Cd(II), and (e) Pb(II), and the stripping peak potentials for (b) Zn(II), (d) Cd(II), and (f) Pb(II). The measurements were performed using 1.31Bi1.08Sn0.44Sb in 0.1 M acetate buffer. Figure 9g) shows the increase in stripping peaks with increasing concentration of the analytes (simultaneously). The full symbols in Figure (a,c,e) characterize the linear concentration range, whereas the empty symbols characterize concentrations above and below the linear concentration range. **Figure S12**: Linear concentration ranges for (a) Zn(II), (c) Cd(II), and (e) Pb(II), and the stripping peak potentials for (b) Zn(II), (d) Cd(II), and (f) Pb(II). The measurements were performed using 1.19Bi0.60Sn0.39Sb in 0.1 M acetate buffer. Figure (g) shows the increase in stripping peaks with increasing concentration of the analytes (simultaneously). The full symbols in Figure (a,c,e) characterize the linear concentration range, whereas the empty symbols characterize concentrations above and below the linear concentration range. **Figure S13**: Linear concentration ranges for (a) Zn(II), (c) Cd(II), and (e) Pb(II), and the stripping peak potentials for (b) Zn(II), (d) Cd(II), and (f) Pb(II). The measurements were performed using 1.15Bi1.22Sn0.57Sb in 0.1 M acetate buffer. Figure (g) shows the increase in stripping peaks with increasing concentration of the analytes (simultaneously). The full symbols in Figure (a,c,e) characterize the linear concentration range, whereas the empty symbols characterize concentrations above and below the linear concentration range. **Figure S14**: Linear concentration ranges for (a) Zn(II), (c) Cd(II), and (e) Pb(II), and the stripping peak potentials for (b) Zn(II), (d) Cd(II), and (f) Pb(II). The measurements were performed using 0.58Bi0.49Sn0.38Sb in 0.1 M acetate buffer. Figure (g) shows the increase in stripping peaks with increasing concentration of the analytes (simultaneously). The full symbols in Figure (a,c,e) characterize the linear concentration range, whereas the empty symbols characterize concentrations above and below the linear concentration range. **Figure S15**: Linear concentration ranges for (a) Zn(II), (c) Cd(II), and (e) Pb(II), and the stripping peak potentials for (b) Zn(II), (d) Cd(II), and (f) Pb(II). The measurements were performed using 1.00Bi0.79Sn in 0.1 M acetate buffer. Figure (g) shows the increase in stripping peaks with increasing concentration of the analytes (simultaneously). The full symbols in Figure (a,c,e) characterize the linear concentration range, whereas the empty symbols characterize concentrations above and below the linear concentration range. **Figure S16**: Linear concentration ranges for (a) Zn(II), (c) Cd(II), and (e) Pb(II), and the stripping peak potentials for (b) Zn(II), (d) Cd(II), and (f) Pb(II). The measurements were performed using 1.31Bi0.64Sn0.64Sb in 0.1 M acetate buffer. Figure (g) shows the increase in stripping peaks with increasing concentration of the analytes (simultaneously). The full symbols in Figure (a,c,e) characterize the linear concentration range, whereas the empty symbols characterize concentrations above and below the linear concentration range. **Figure S17**: Linear concentration ranges for (a) Zn(II), (c) Cd(II), and (e) Pb(II), and the stripping peak potentials for (b) Zn(II), (d) Cd(II), and (f) Pb(II). The measurements were performed using 0.59Bi0.10Sn in 0.1 M acetate buffer. Figure g) shows the increase in stripping peaks with increasing concentration of the analytes (simultaneously). The full symbols in Figure (a,c,e) characterize the linear concentration range, whereas the empty symbols characterize concentrations above and below the linear concentration range. **Figure S18**: Linear concentration ranges for (a) Zn(II), (c) Cd(II), and (e) Pb(II), and the stripping peak potentials for (b) Zn(II), (d) Cd(II), and (f) Pb(II). The measurements were performed using 1.08Bi0.19Sn0.41Sb in 0.1 M acetate buffer. Figure (g) shows the increase in stripping peaks with increasing concentration of the analytes (simultaneously). The full symbols in Figure (a,c,e) characterize the linear concentration range, whereas the empty symbols characterize concentrations above and below the linear concentration range. **Figure S19**: Linear concentration ranges for (a) Zn(II), (c) Cd(II), and (e) Pb(II), and the stripping peak potentials for (b) Zn(II), (d) Cd(II), and (f) Pb(II). The measurements were performed using 0.94Bi0.80Sn0.30Sb in 0.1 M acetate buffer. Figure (g) shows the increase in stripping peaks with increasing concentration of the analytes (simultaneously). The full symbols in Figure (a,c,e) characterize the linear concentration range, whereas the empty symbols characterize concentrations above and below the linear concentration range. **Figure S20**: Linear concentration ranges for (a) Zn(II), (c) Cd(II), and (e) Pb(II), and the stripping peak potentials for (b) Zn(II), (d) Cd(II), and (f) Pb(II). The measurements were performed using 0.94Bi0.67Sn0.28Sb in 0.1 M acetate buffer. Figure (g) shows the increase in stripping peaks with increasing concentration of the analytes (simultaneously). The full symbols in Figure (a,c,e) characterize the linear concentration range, whereas the empty symbols characterize concentrations above and below the linear concentration range. **Figure S21**: Linear concentration ranges for (a) Zn(II), (c) Cd(II), and (e) Pb(II), and the stripping peak potentials for (b) Zn(II), (d) Cd(II), and (f) Pb(II). The measurements were performed using 1.31Bi0.72Sn0.23Sb in 0.1 M acetate buffer. Figure (g) shows the increase in stripping peaks with increasing concentration of the analytes (simultaneously). The full symbols in Figure (a,c,e) characterize the linear concentration range, whereas the empty symbols characterize concentrations above and below the linear concentration range.
