# Supplementary material for: The Use of Factorial Design and Simplex Optimization to Improve Analytical Performance of In Situ Film Electrodes
Source: Sensors (Basel). 2020 Jul 14;20(14):3921. doi: 10.3390/s20143921 (PMC7411898; doi:10.3390/s20143921)
Supplement: Supplementary file 1 [file sensors-20-03921-s001.pdf]

Supplementary material

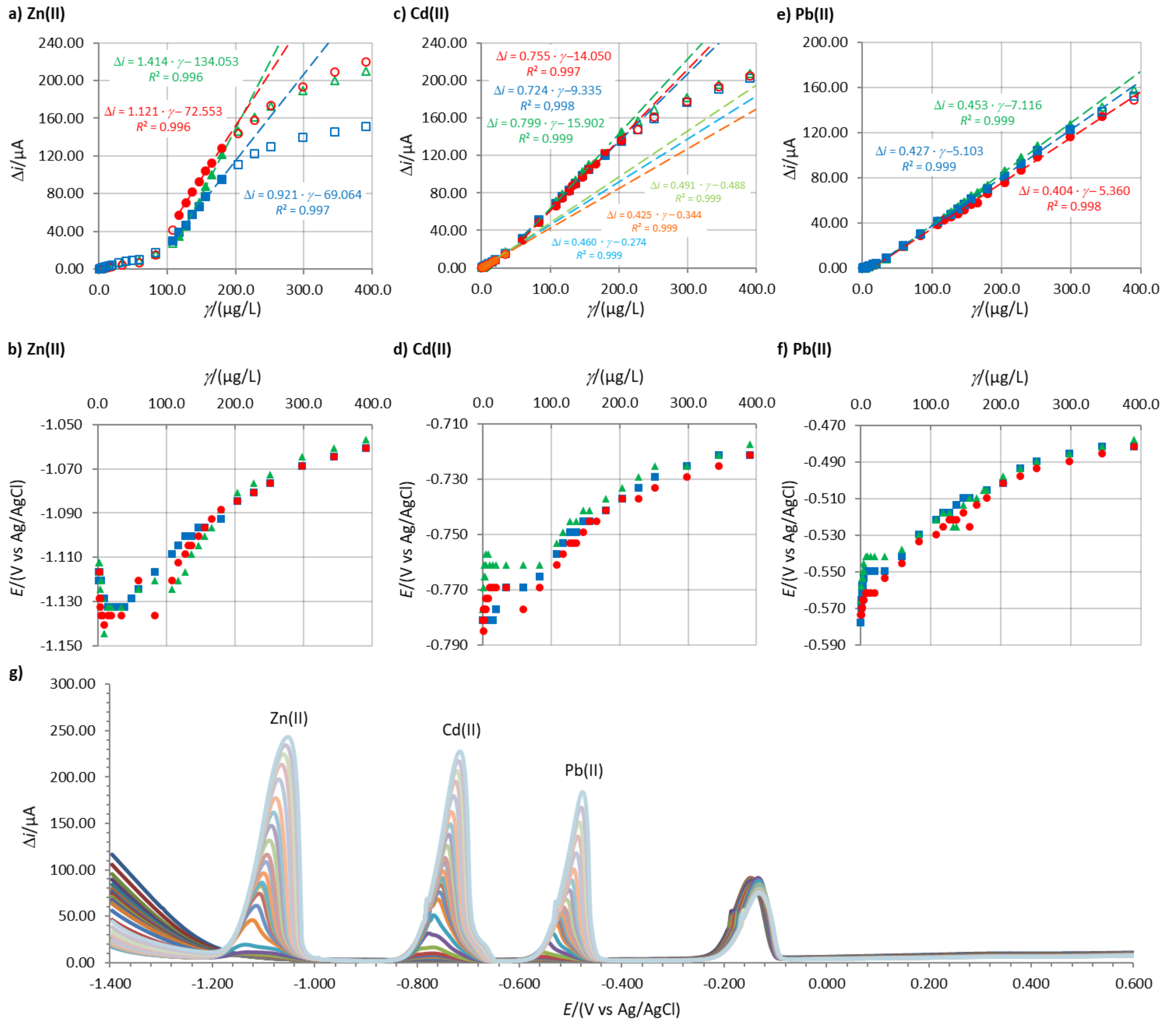

Figure S1: Linear concentration ranges for a) Zn(II), c) Cd(II), and e) Pb(II), and the stripping peak potentials for b) Zn(II), d) Cd(II), and f) Pb(II). The measurements were performed using 0.80Bi0.70Sn0.80Sb in 0.1 M acetate buffer. Figure g) shows the increase in stripping peaks with increasing concentration of the analytes (simultaneously). The full symbols in Figure a,c,e) characterize the linear concentration range, whereas the empty symbols characterize concentrations above and below the linear concentration range.

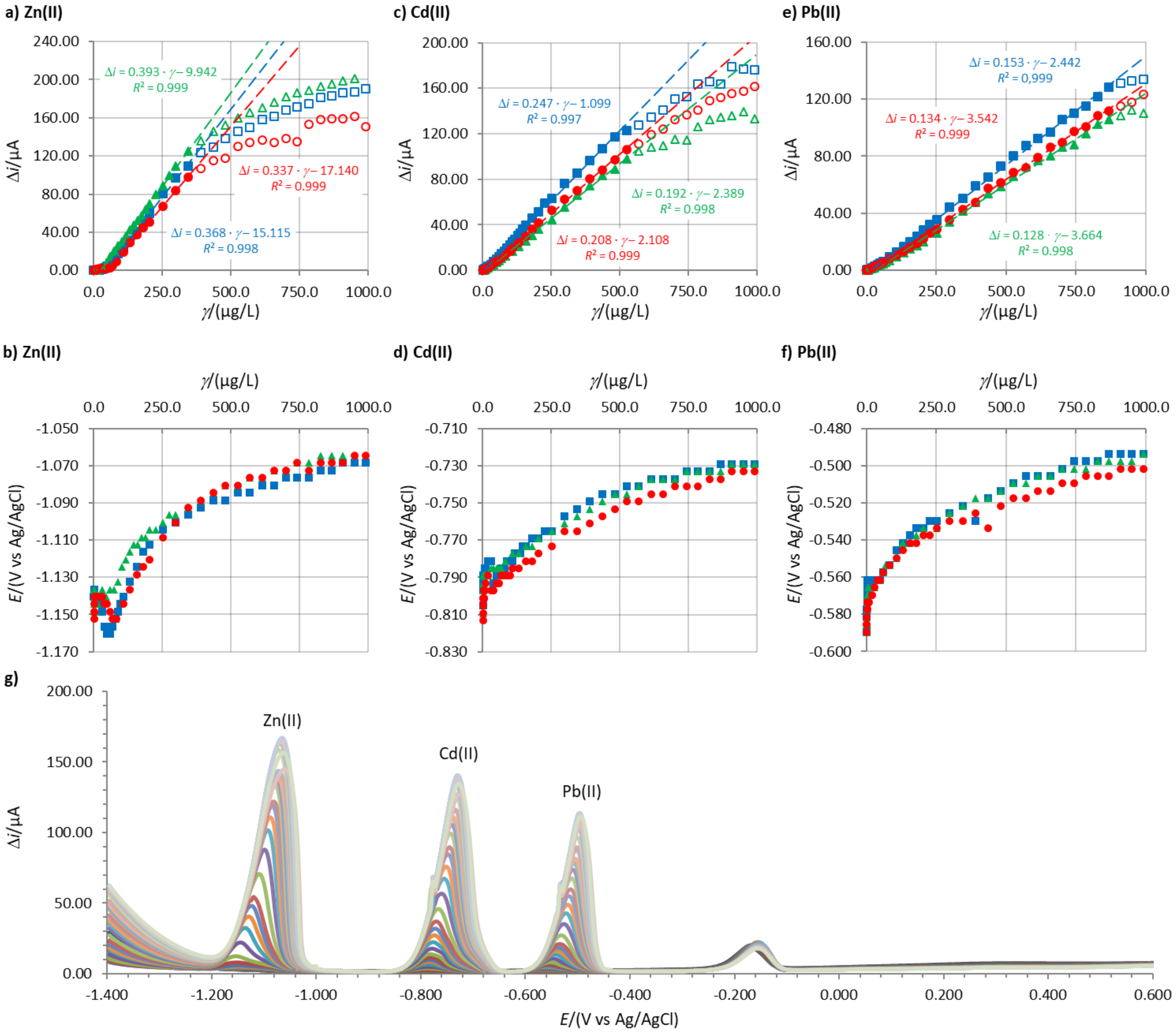

Figure S2: Linear concentration ranges for a) Zn(II), c) Cd(II), and e) Pb(II), and the stripping peak potentials for b) Zn(II), d) Cd(II), and f) Pb(II). The measurements were performed using 0.60Bi0.80Sn0.30Sb in 0.1 M acetate buffer. Figure g) shows the increase in stripping peaks with increasing concentration of the analytes (simultaneously). The full symbols in Figure a,c,e) characterize the linear concentration range, whereas the empty symbols characterize concentrations above and below the linear concentration range.

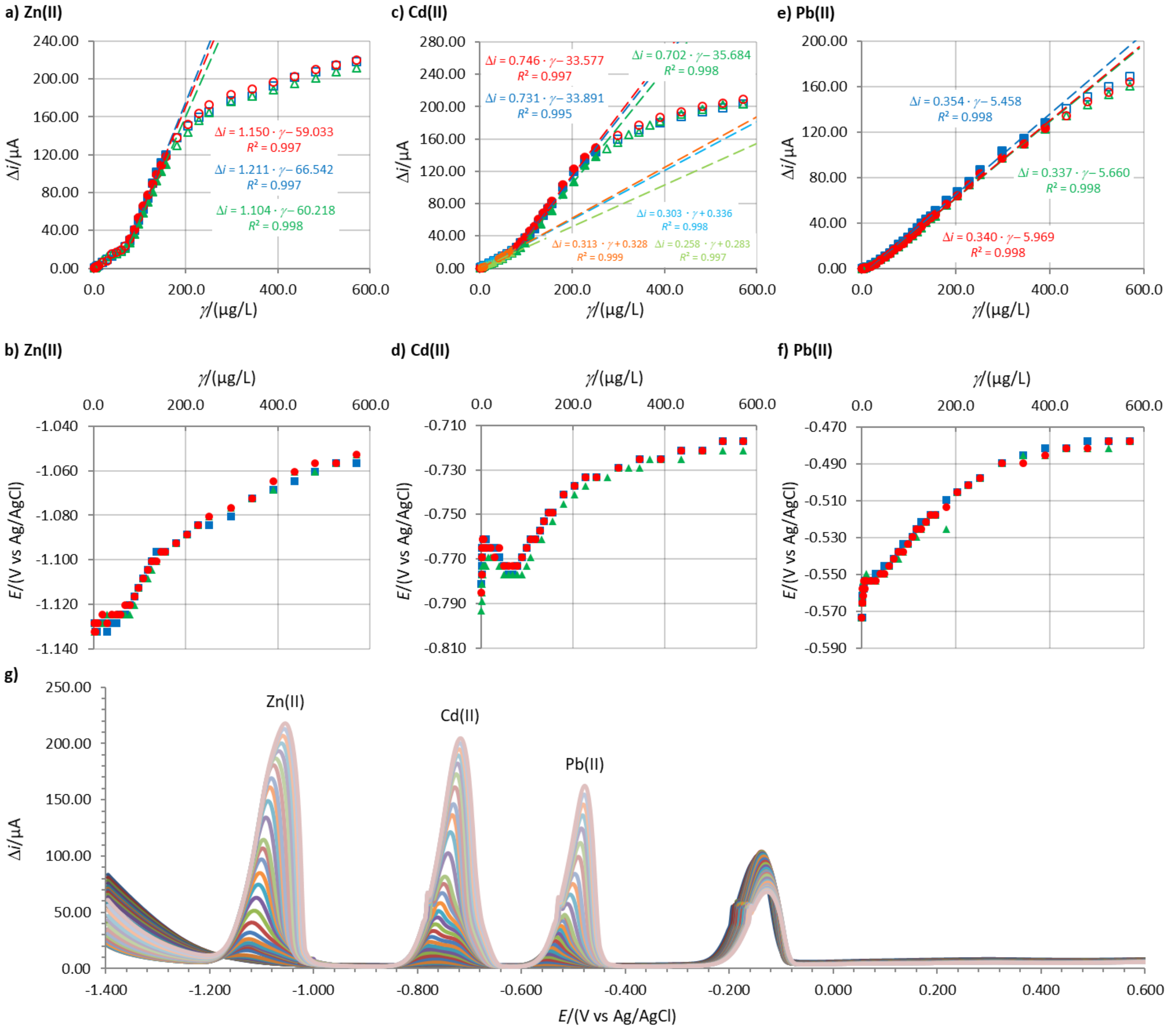

Figure S3: Linear concentration ranges for a) Zn(II), c) Cd(II), and e) Pb(II), and the stripping peak potentials for b) Zn(II), d) Cd(II), and f) Pb(II). The measurements were performed using 0.70Bi0.20Sn0.70Sb in 0.1 M acetate buffer. Figure g) shows the increase in stripping peaks with increasing concentration of the analytes (simultaneously). The full symbols in Figure a,c,e) characterize the linear concentration range, whereas the empty symbols characterize concentrations above and below the linear concentration range.

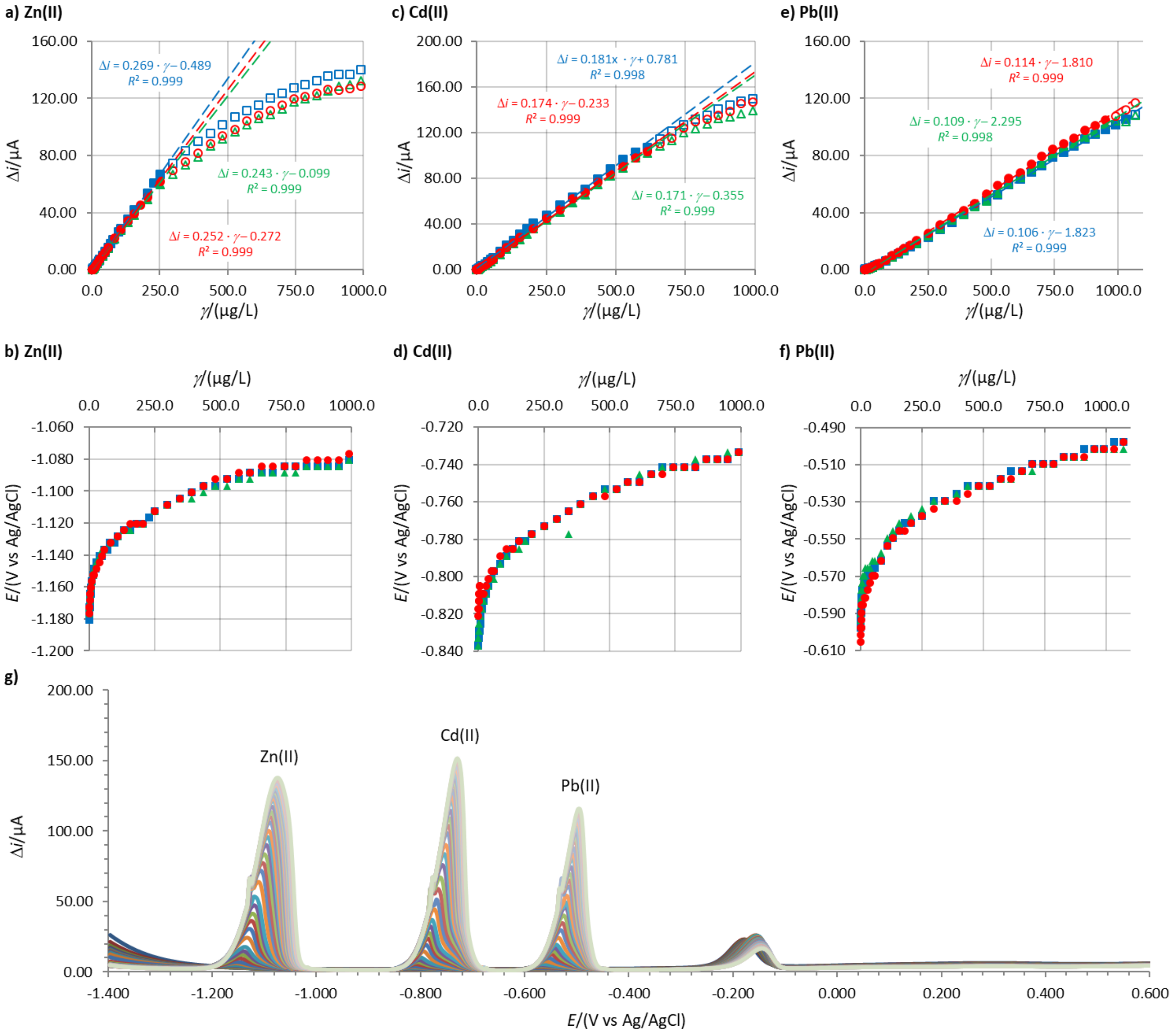

Figure S4: Linear concentration ranges for a) Zn(II), c) Cd(II), and e) Pb(II), and the stripping peak potentials for b) Zn(II), d) Cd(II), and f) Pb(II). The measurements were performed using 0.80Bi0.30Sn0.20Sb in 0.1 M acetate buffer. Figure g) shows the increase in stripping peaks with increasing concentration of the analytes (simultaneously). The full symbols in Figure a,c,e) characterize the linear concentration range, whereas the empty symbols characterize concentrations above and below the linear concentration range.

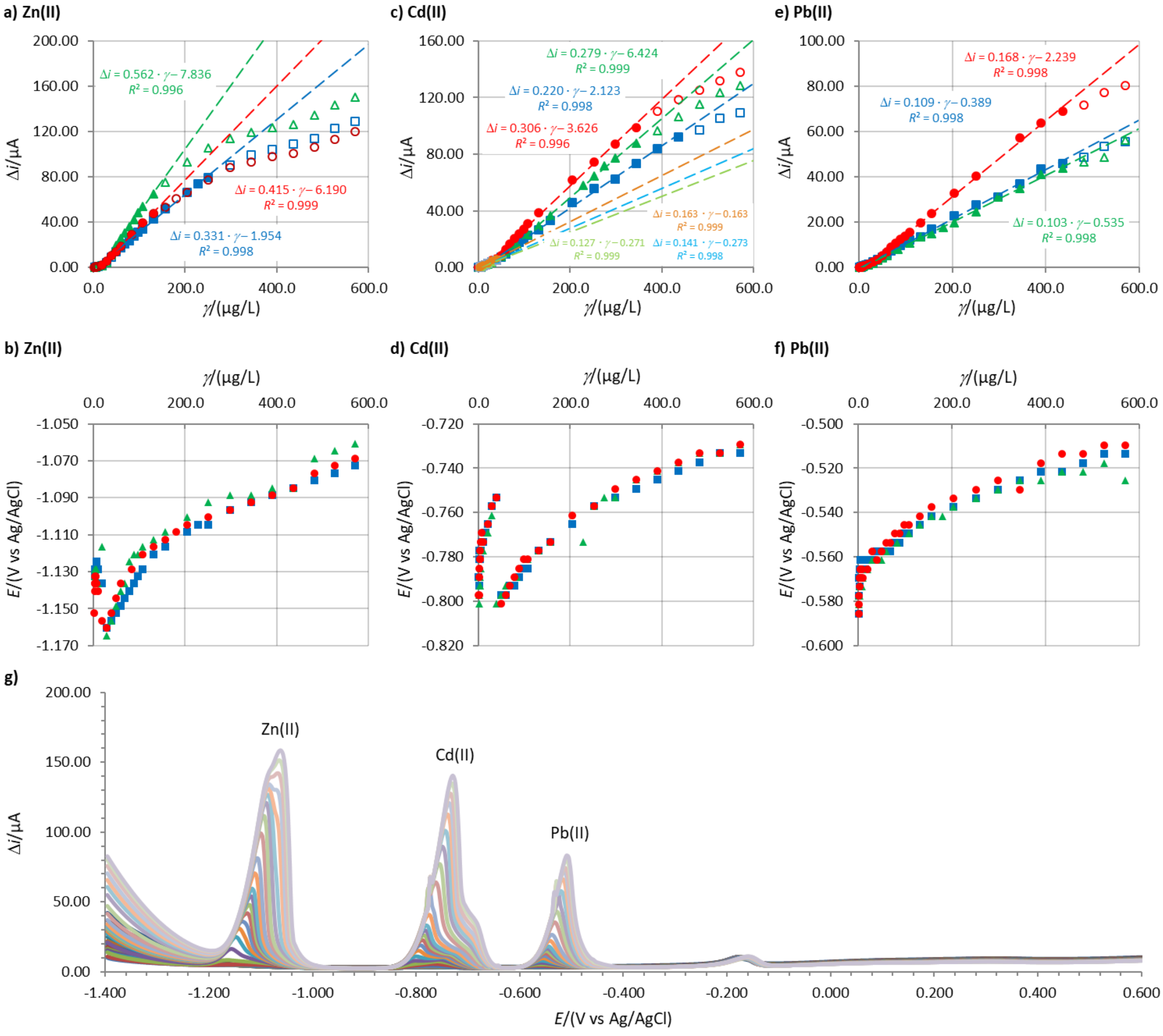

Figure S5: Linear concentration ranges for a) Zn(II), c) Cd(II), and e) Pb(II), and the stripping peak potentials for b) Zn(II), d) Cd(II), and f) Pb(II). The measurements were performed using 0.20Bi0.80Sn0.80Sb in 0.1 M acetate buffer. Figure g) shows the increase in stripping peaks with increasing concentration of the analytes (simultaneously). The full symbols in Figure a,c,e) characterize the linear concentration range, whereas the empty symbols characterize concentrations above and below the linear concentration range.

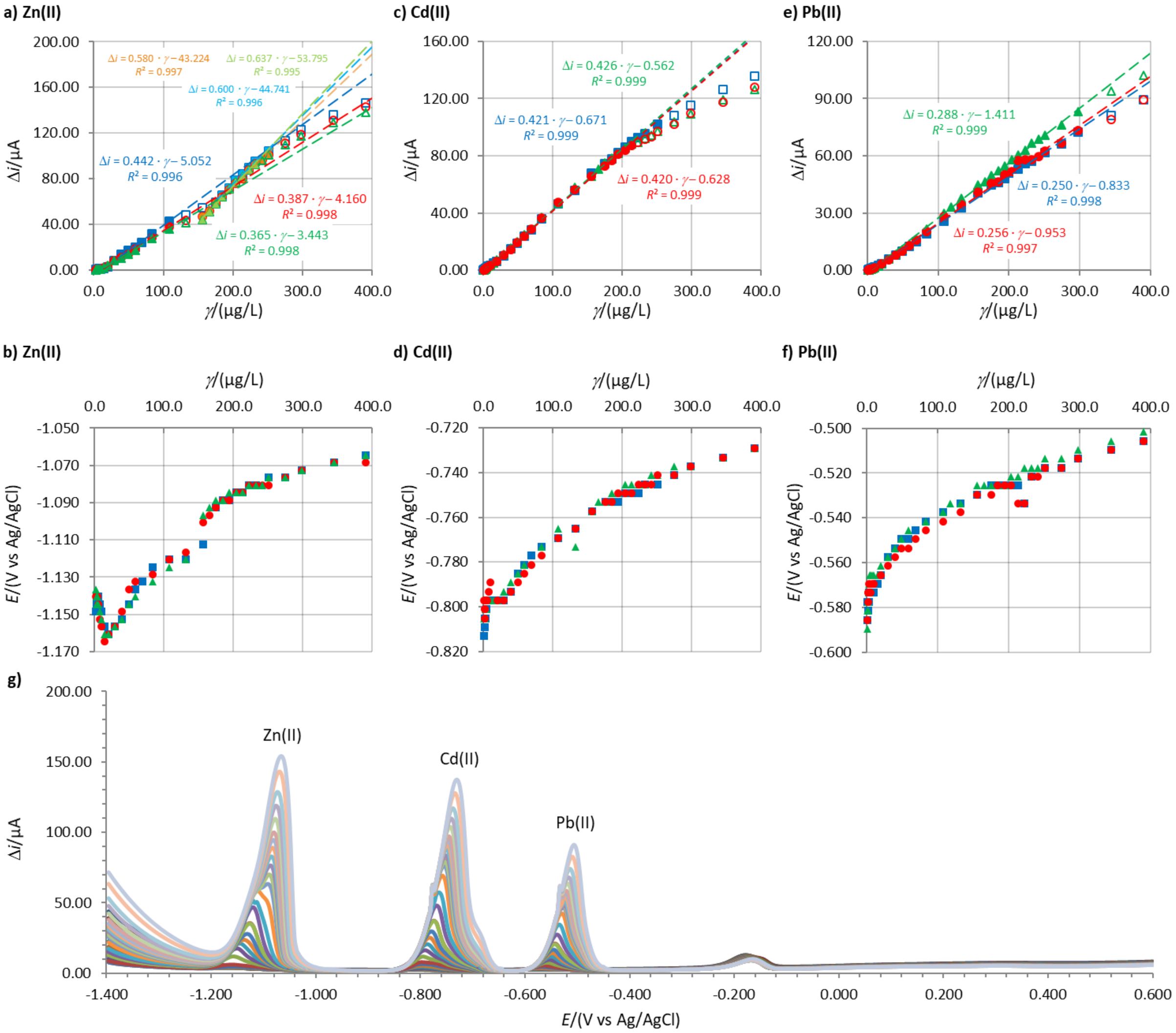

Figure S6: Linear concentration ranges for a) Zn(II), c) Cd(II), and e) Pb(II), and the stripping peak potentials for b) Zn(II), d) Cd(II), and f) Pb(II). The measurements were performed using 0.20Bi0.70Sn0.30Sb in 0.1 M acetate buffer. Figure g) shows the increase in stripping peaks with increasing concentration of the analytes (simultaneously). The full symbols in Figure a,c,e) characterize the linear concentration range, whereas the empty symbols characterize concentrations above and below the linear concentration range.

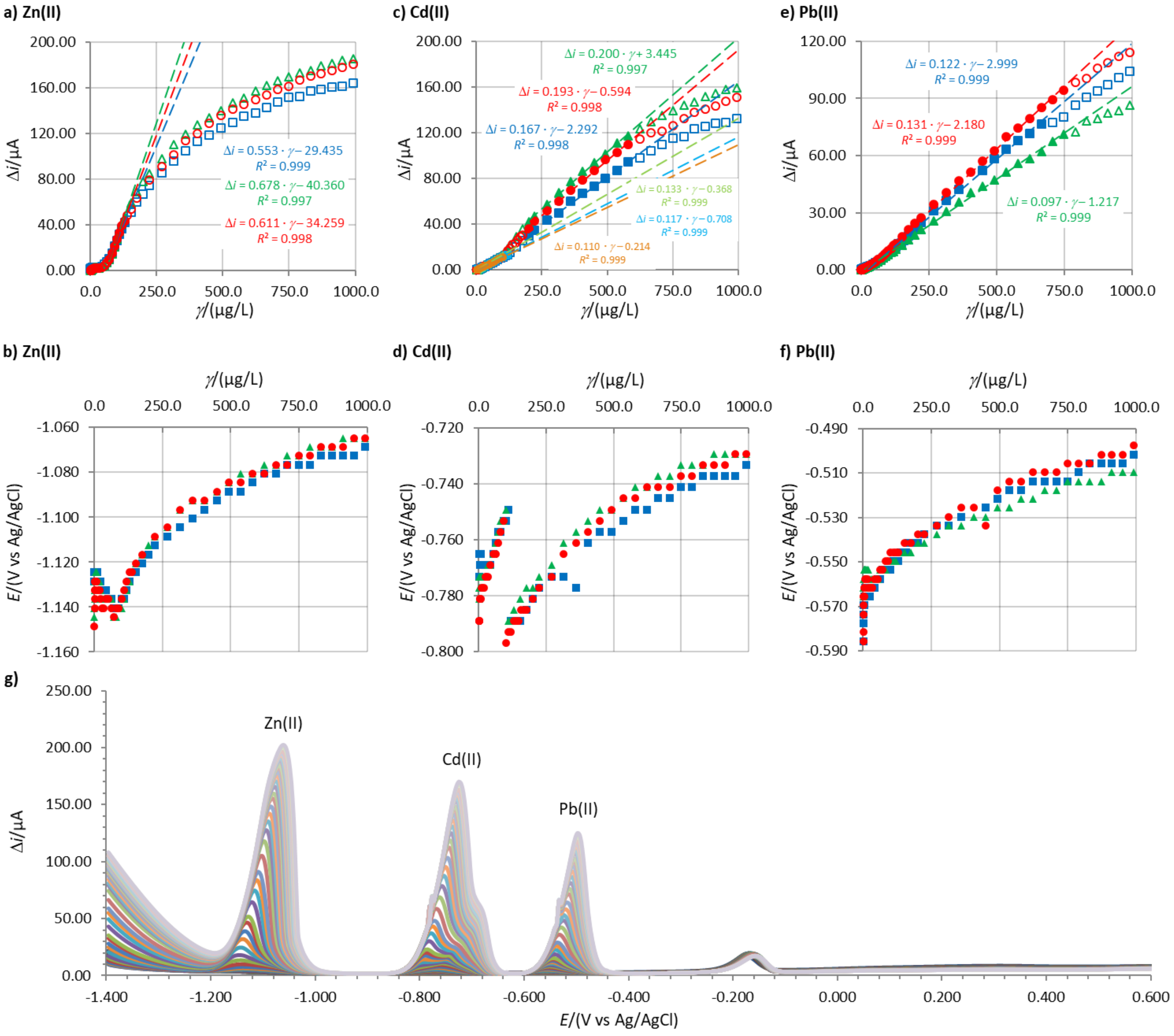

Figure S7: Linear concentration ranges for a) Zn(II), c) Cd(II), and e) Pb(II), and the stripping peak potentials for b) Zn(II), d) Cd(II), and f) Pb(II). The measurements were performed using 0.30Bi0.30Sn0.60Sb in 0.1 M acetate buffer. Figure g) shows the increase in stripping peaks with increasing concentration of the analytes (simultaneously). The full symbols in Figure a,c,e) characterize the linear concentration range, whereas the empty symbols characterize concentrations above and below the linear concentration range.

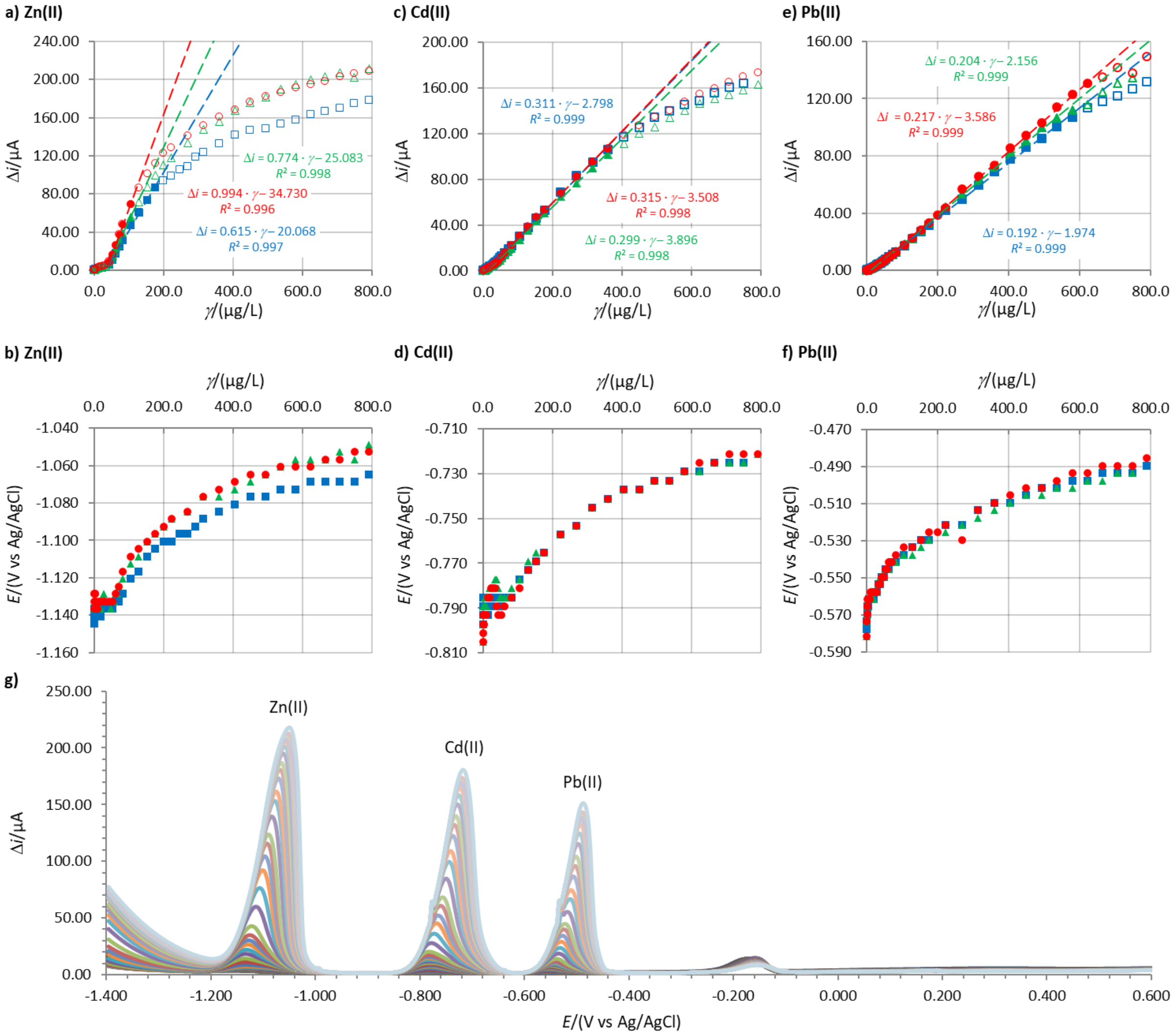

Figure S8: Linear concentration ranges for a) Zn(II), c) Cd(II), and e) Pb(II), and the stripping peak potentials for b) Zn(II), d) Cd(II), and f) Pb(II). The measurements were performed using 0.20Bi0.20Sn0.20Sb in 0.1 M acetate buffer. Figure g) shows the increase in stripping peaks with increasing concentration of the analytes (simultaneously). The full symbols in Figure a,c,e) characterize the linear concentration range, whereas the empty symbols characterize concentrations above and below the linear concentration range.

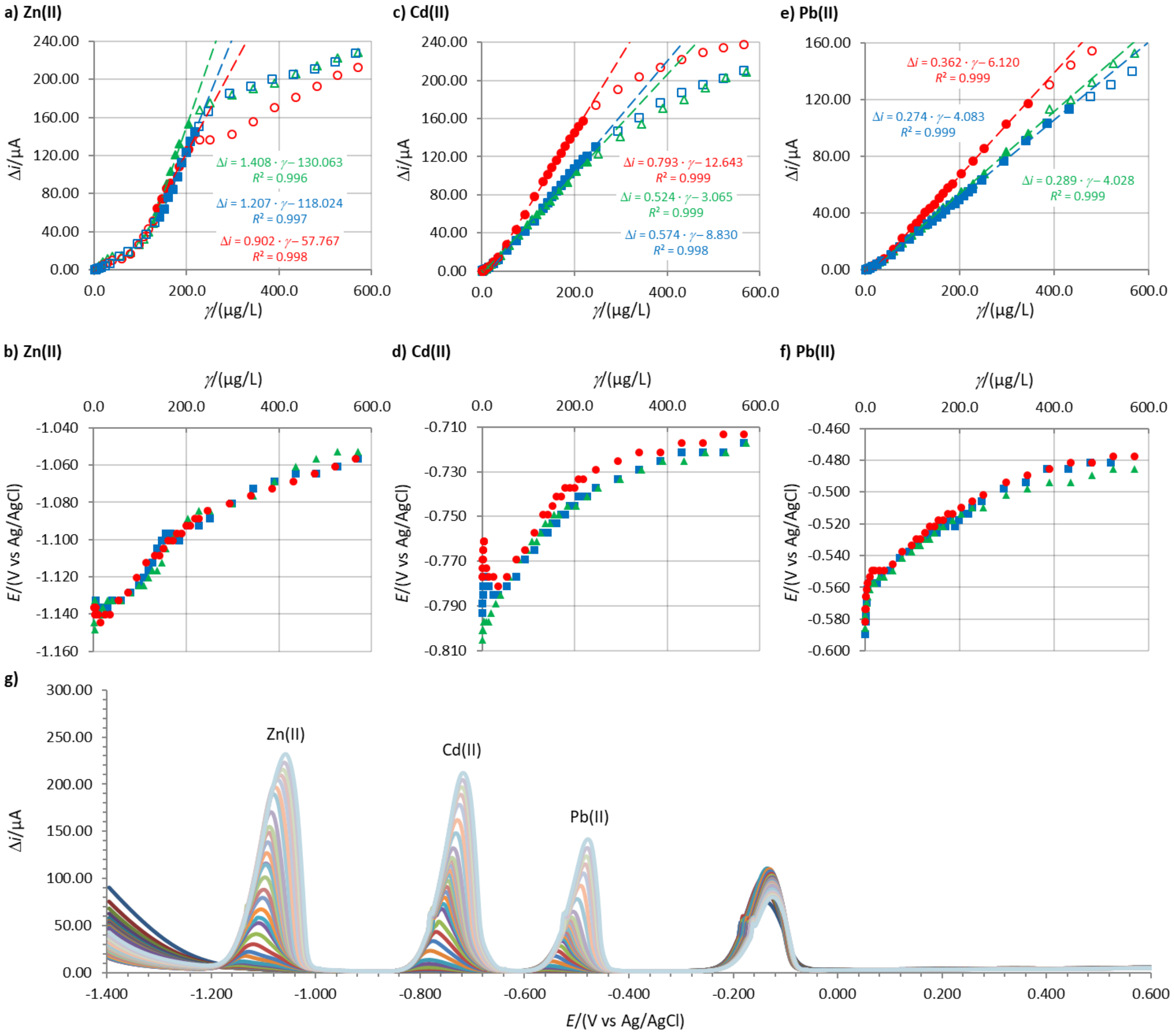

Figure S9: Linear concentration ranges for a) Zn(II), c) Cd(II), and e) Pb(II), and the stripping peak potentials for b) Zn(II), d) Cd(II), and f) Pb(II). The measurements were performed using 0.94Bi0.58Sn0.28Sb in 0.1 M acetate buffer. Figure g) shows the increase in stripping peaks with increasing concentration of the analytes (simultaneously). The full symbols in Figure a,c,e) characterize the linear concentration range, whereas the empty symbols characterize concentrations above and below the linear concentration range.

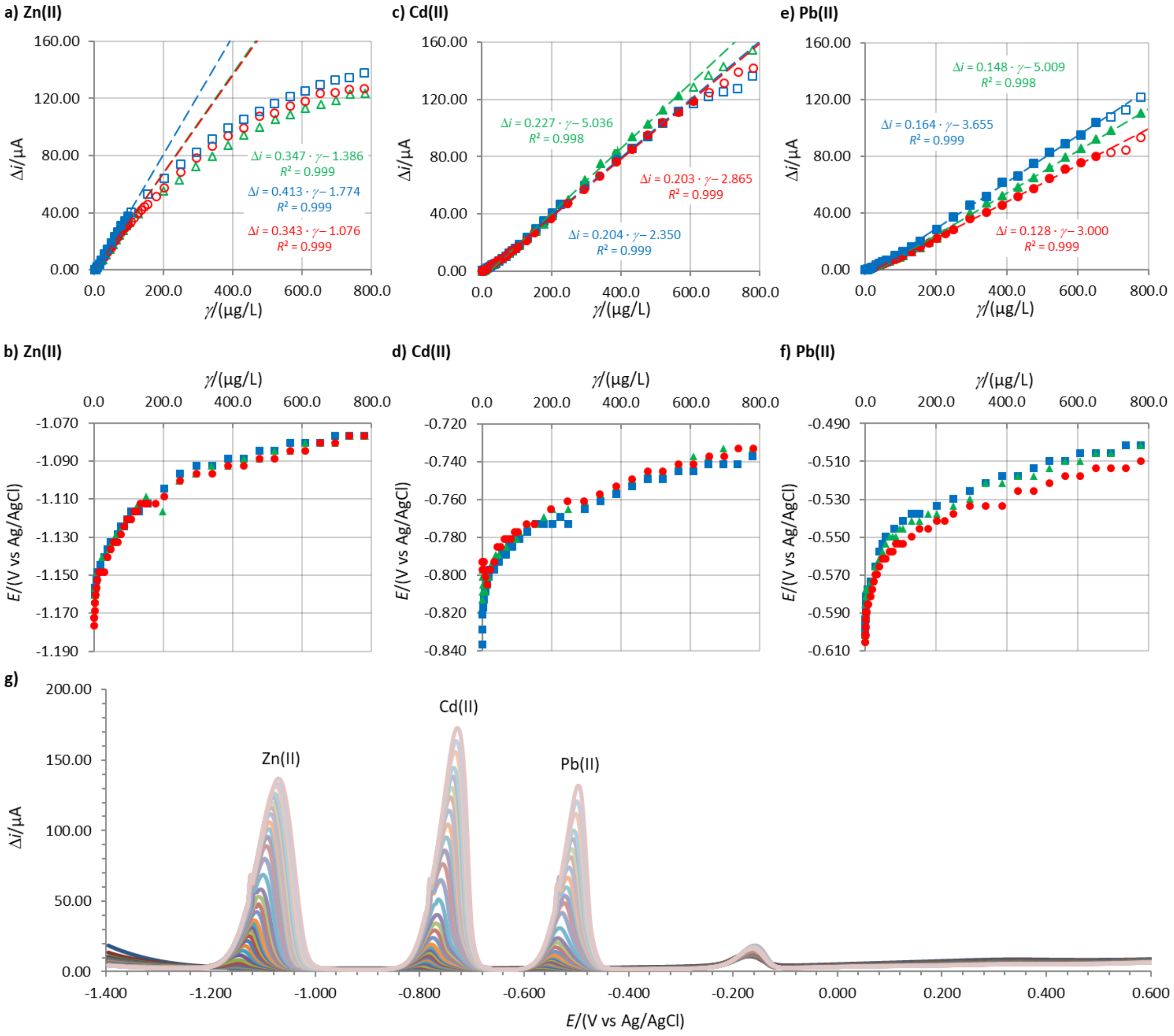

Figure S10: Linear concentration ranges for a) Zn(II), c) Cd(II), and e) Pb(II), and the stripping peak potentials for b) Zn(II), d) Cd(II), and f) Pb(II). The measurements were performed using 0.64Bi0.83Sn0.01Sb in 0.1 M acetate buffer. Figure g) shows the increase in stripping peaks with increasing concentration of the analytes (simultaneously). The full symbols in Figure a,c,e) characterize the linear concentration range, whereas the empty symbols characterize concentrations above and below the linear concentration range.

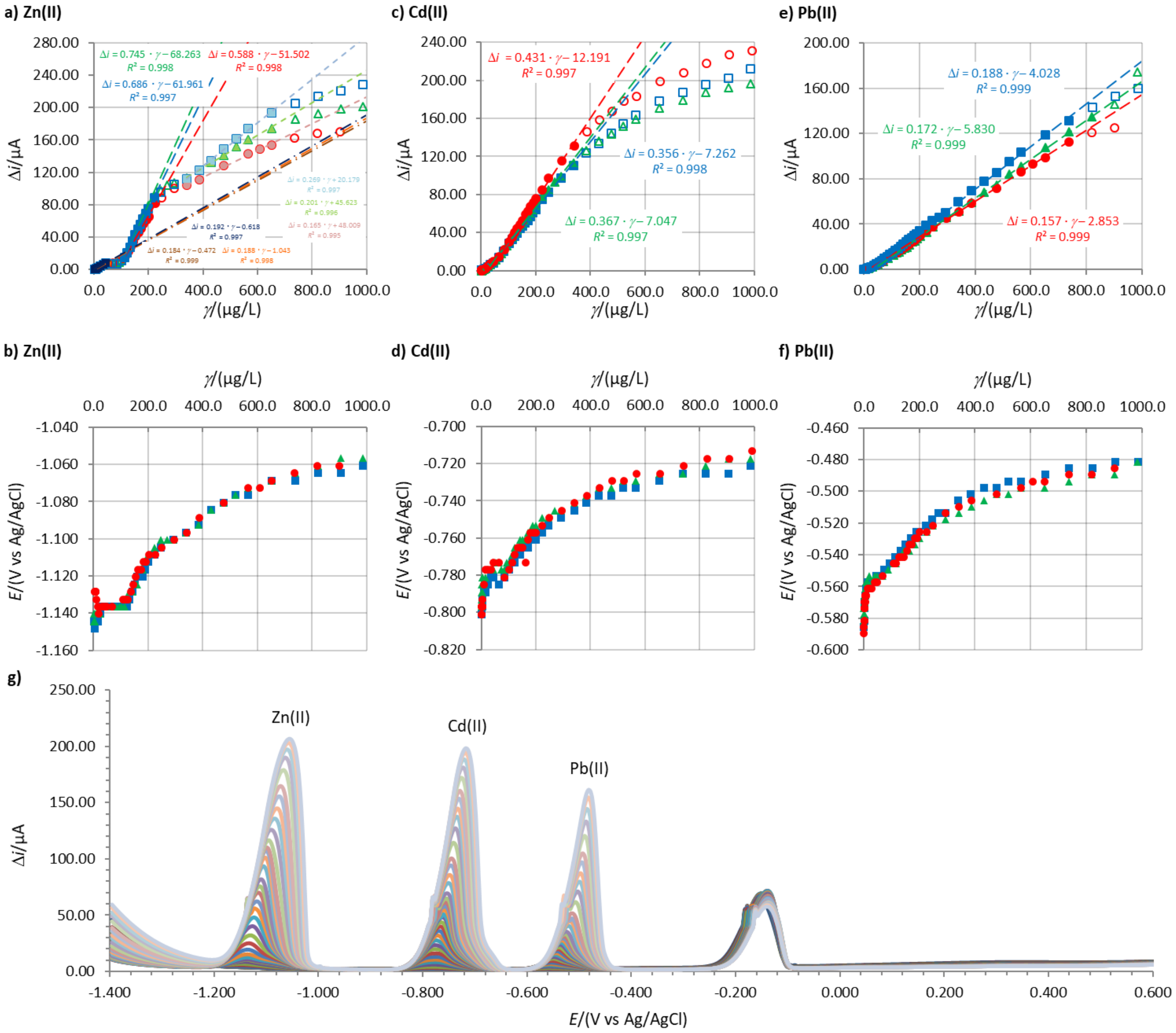

Figure S11: Linear concentration ranges for a) Zn(II), c) Cd(II), and e) Pb(II), and the stripping peak potentials for b) Zn(II), d) Cd(II), and f) Pb(II). The measurements were performed using 1.31Bi1.08Sn0.44Sb in 0.1 M acetate buffer. Figure g) shows the increase in stripping peaks with increasing concentration of the analytes (simultaneously). The full symbols in Figure a,c,e) characterize the linear concentration range, whereas the empty symbols characterize concentrations above and below the linear concentration range.

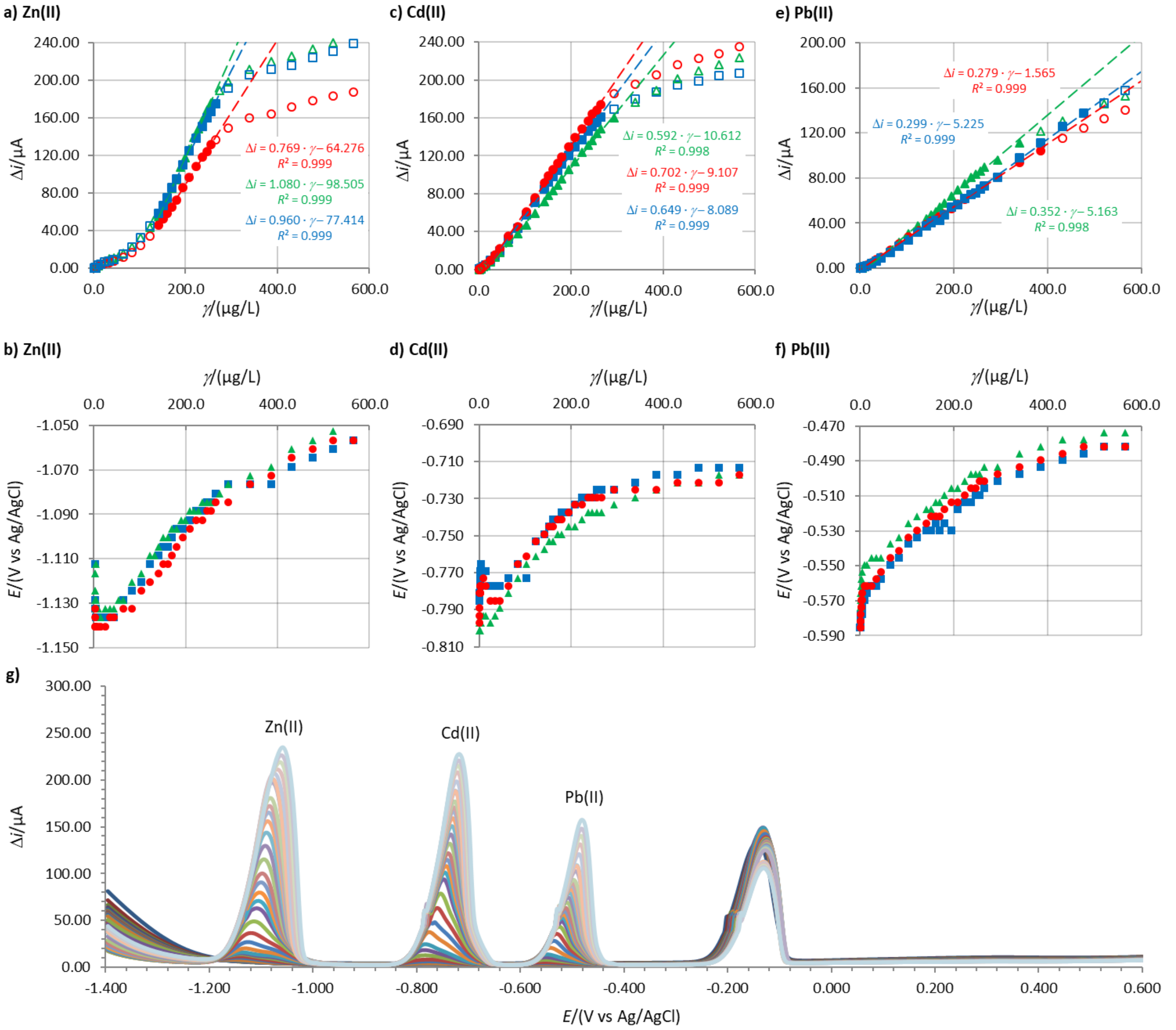

Figure S12: Linear concentration ranges for a) Zn(II), c) Cd(II), and e) Pb(II), and the stripping peak potentials for b) Zn(II), d) Cd(II), and f) Pb(II). The measurements were performed using 1.19Bi0.60Sn0.39Sb in 0.1 M acetate buffer. Figure g) shows the increase in stripping peaks with increasing concentration of the analytes (simultaneously). The full symbols in Figure a,c,e) characterize the linear concentration range, whereas the empty symbols characterize concentrations above and below the linear concentration range.

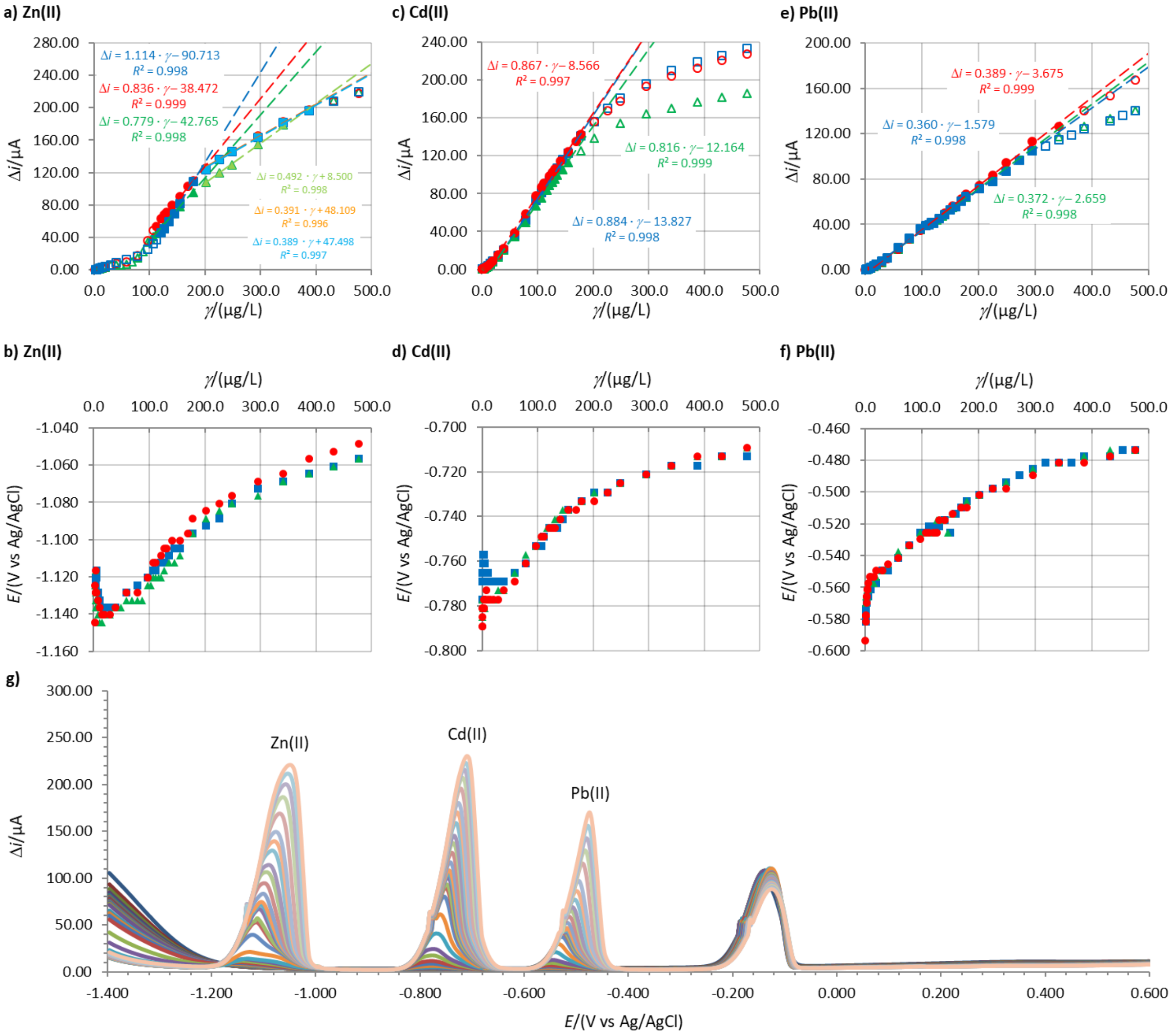

Figure S13: Linear concentration ranges for a) Zn(II), c) Cd(II), and e) Pb(II), and the stripping peak potentials for b) Zn(II), d) Cd(II), and f) Pb(II). The measurements were performed using 1.15Bi1.22Sn0.57Sb in 0.1 M acetate buffer. Figure g) shows the increase in stripping peaks with increasing concentration of the analytes (simultaneously). The full symbols in Figure a,c,e) characterize the linear concentration range, whereas the empty symbols characterize concentrations above and below the linear concentration range.

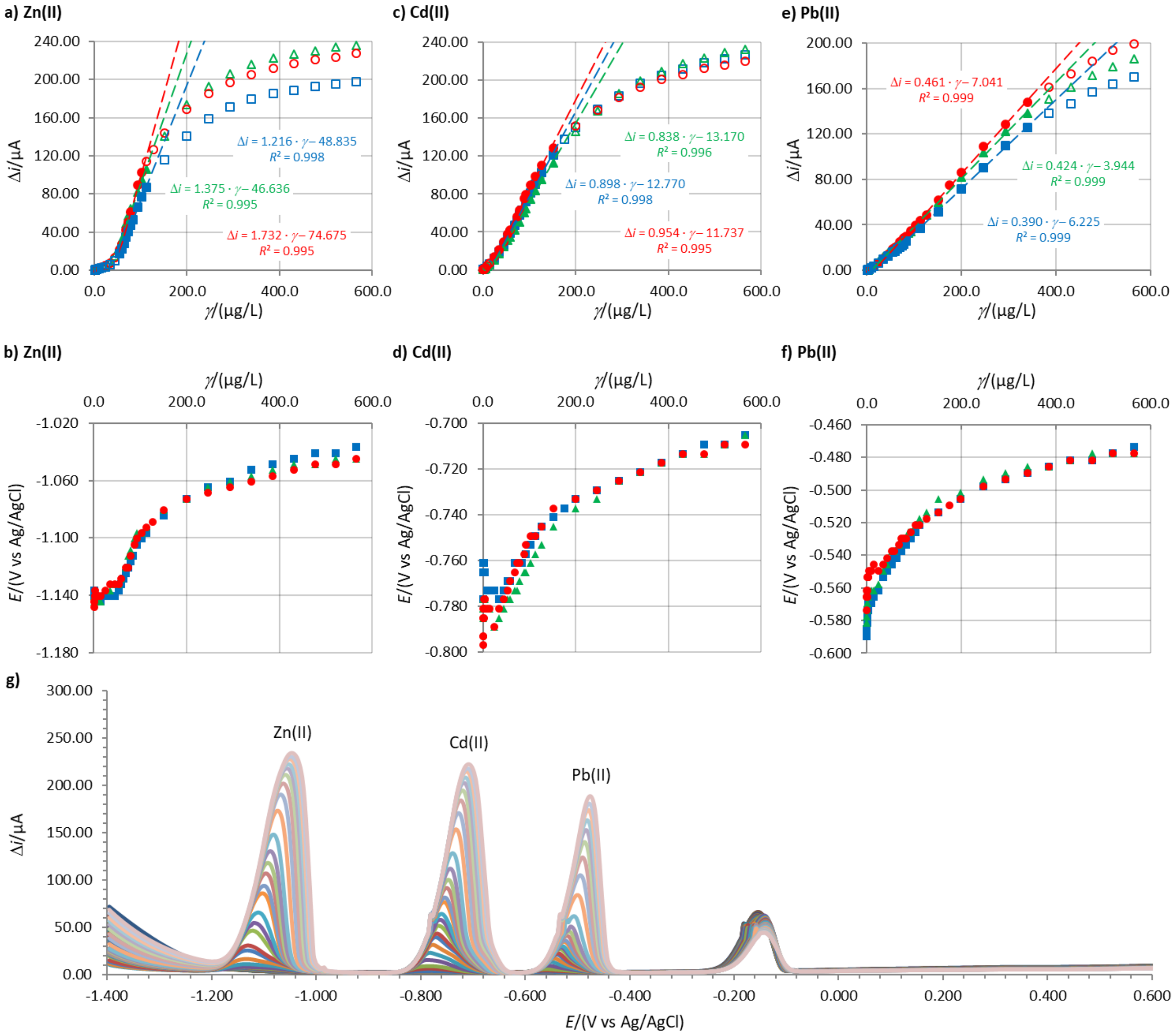

Figure S14: Linear concentration ranges for a) Zn(II), c) Cd(II), and e) Pb(II), and the stripping peak potentials for b) Zn(II), d) Cd(II), and f) Pb(II). The measurements were performed using 0.58Bi0.49Sn0.38Sb in 0.1 M acetate buffer. Figure g) shows the increase in stripping peaks with increasing concentration of the analytes (simultaneously). The full symbols in Figure a,c,e) characterize the linear concentration range, whereas the empty symbols characterize concentrations above and below the linear concentration range.

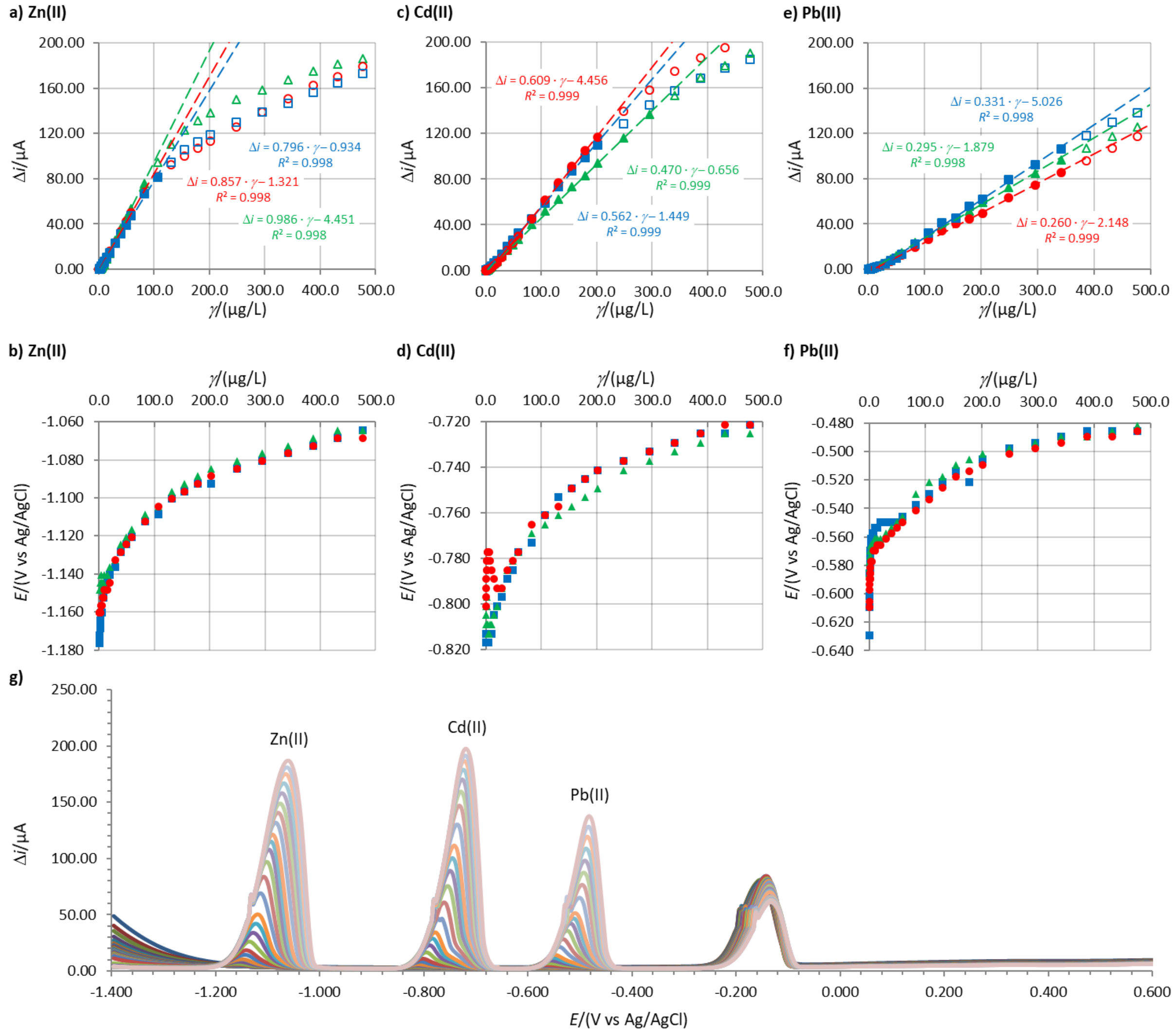

Figure S15: Linear concentration ranges for a) Zn(II), c) Cd(II), and e) Pb(II), and the stripping peak potentials for b) Zn(II), d) Cd(II), and f) Pb(II). The measurements were performed using 1.00Bi0.79Sn in 0.1 M acetate buffer. Figure g) shows the increase in stripping peaks with increasing concentration of the analytes (simultaneously). The full symbols in Figure a,c,e) characterize the linear concentration range, whereas the empty symbols characterize concentrations above and below the linear concentration range.

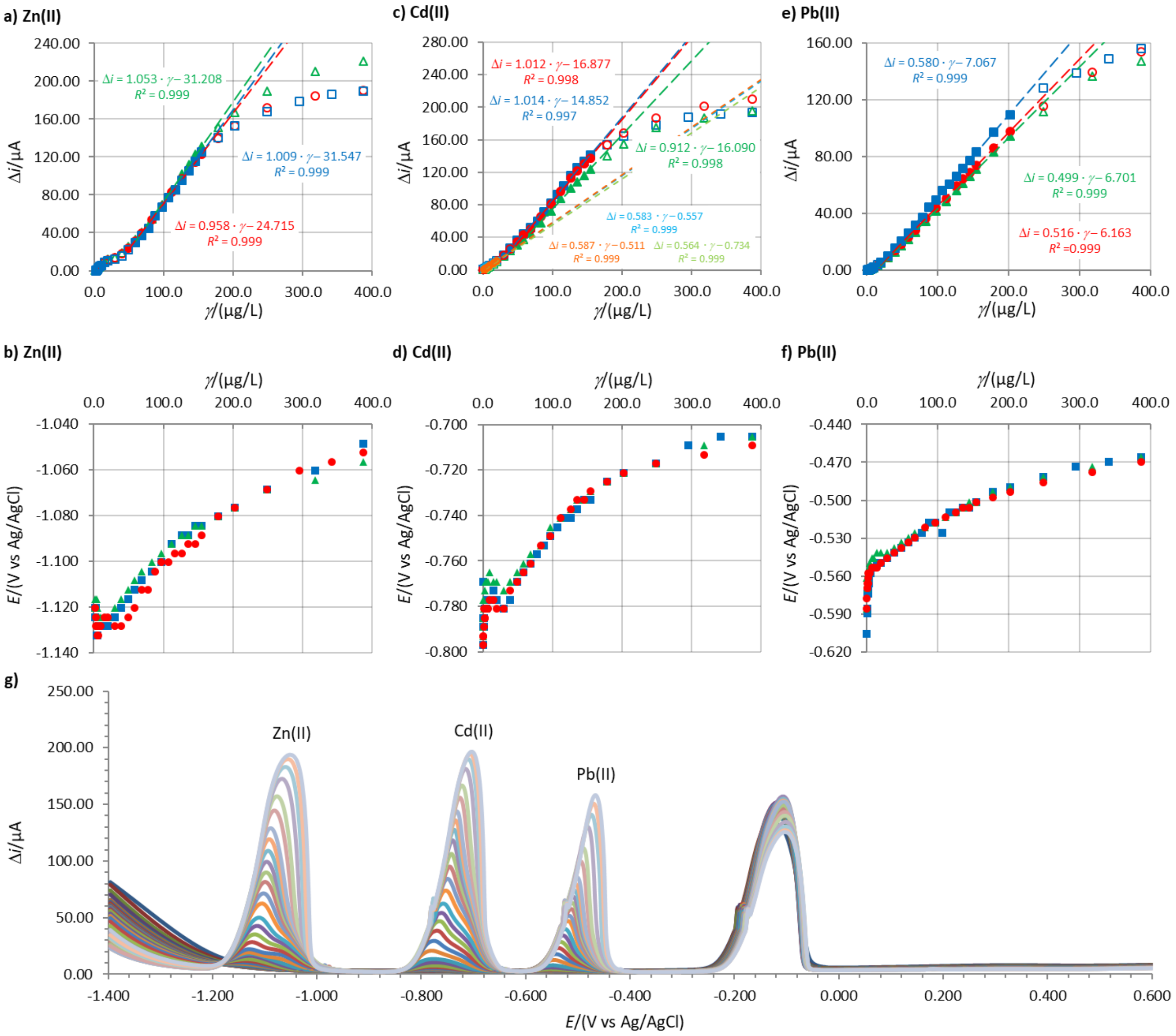

Figure S16: Linear concentration ranges for a) Zn(II), c) Cd(II), and e) Pb(II), and the stripping peak potentials for b) Zn(II), d) Cd(II), and f) Pb(II). The measurements were performed using 1.31Bi0.64Sn0.64Sb in 0.1 M acetate buffer. Figure g) shows the increase in stripping peaks with increasing concentration of the analytes (simultaneously). The full symbols in Figure a,c,e) characterize the linear concentration range, whereas the empty symbols characterize concentrations above and below the linear concentration range.

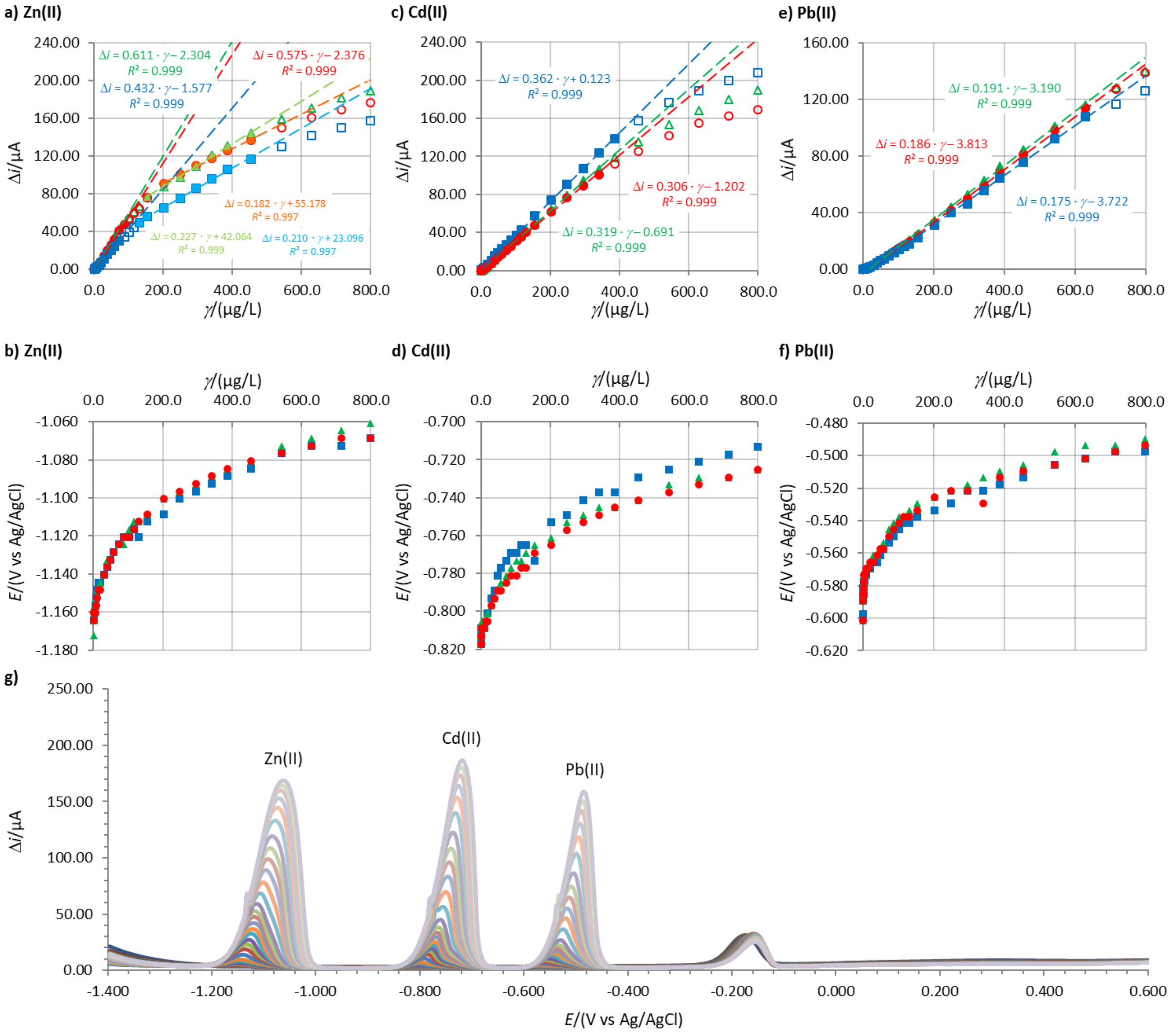

Figure S17: Linear concentration ranges for a) Zn(II), c) Cd(II), and e) Pb(II), and the stripping peak potentials for b) Zn(II), d) Cd(II), and f) Pb(II). The measurements were performed using 0.59Bi0.10Sn in 0.1 M acetate buffer. Figure g) shows the increase in stripping peaks with increasing concentration of the analytes (simultaneously). The full symbols in Figure a,c,e) characterize the linear concentration range, whereas the empty symbols characterize concentrations above and below the linear concentration range.

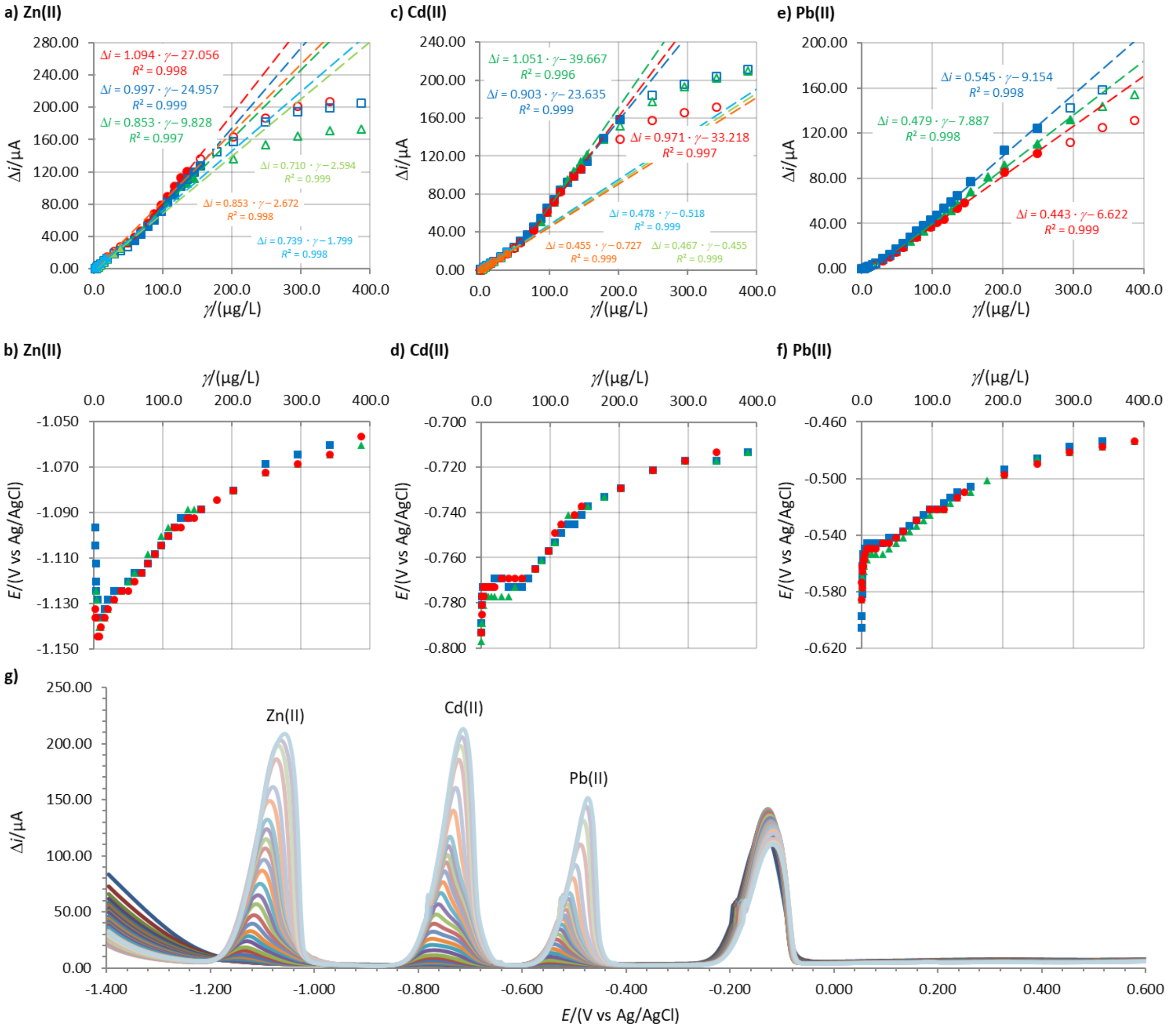

Figure S18: Linear concentration ranges for a) Zn(II), c) Cd(II), and e) Pb(II), and the stripping peak potentials for b) Zn(II), d) Cd(II), and f) Pb(II). The measurements were performed using 1.08Bi0.19Sn0.41Sb in 0.1 M acetate buffer. Figure g) shows the increase in stripping peaks with increasing concentration of the analytes (simultaneously). The full symbols in Figure a,c,e) characterize the linear concentration range, whereas the empty symbols characterize concentrations above and below the linear concentration range.

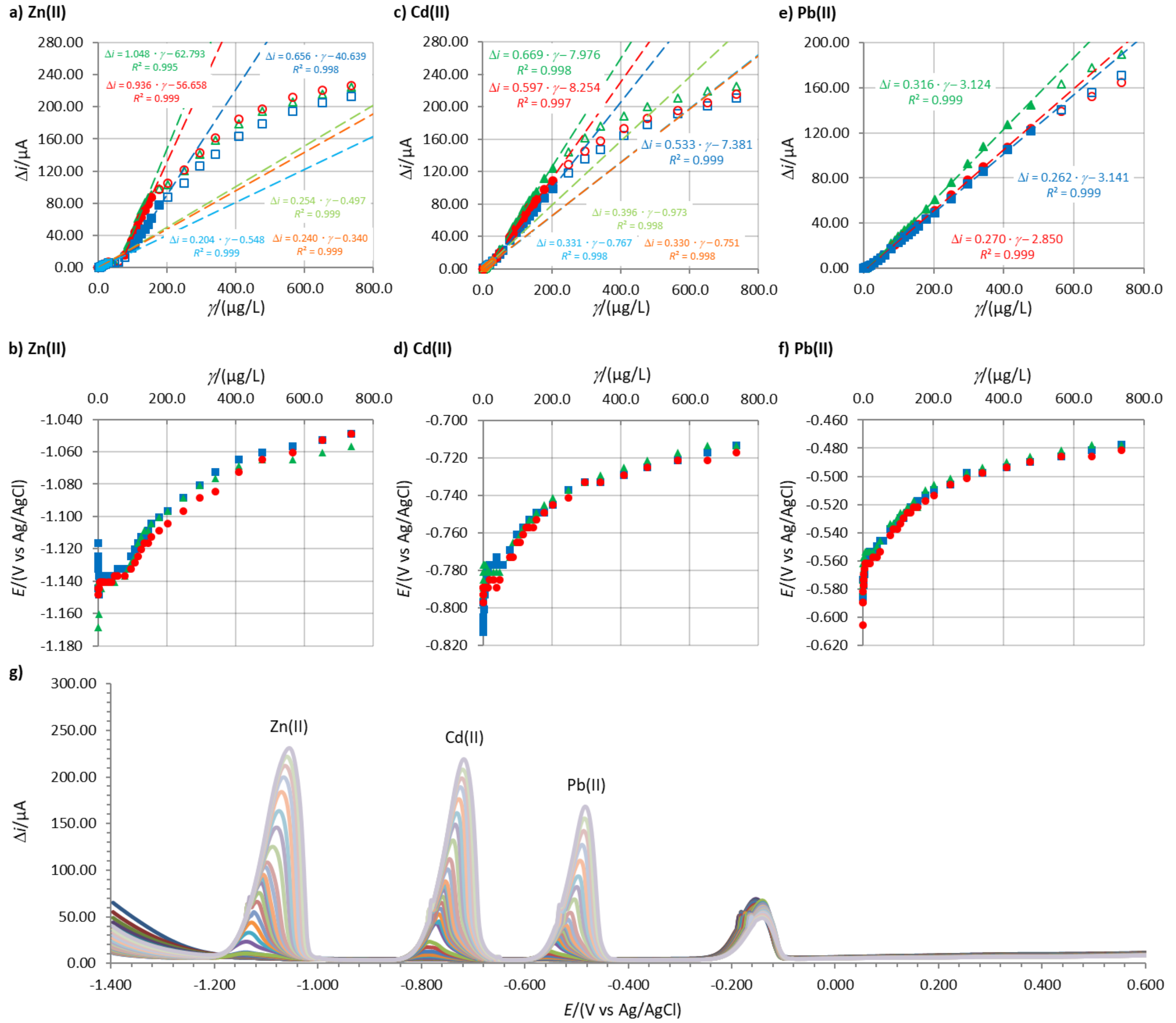

Figure S19: Linear concentration ranges for a) Zn(II), c) Cd(II), and e) Pb(II), and the stripping peak potentials for b) Zn(II), d) Cd(II), and f) Pb(II). The measurements were performed using 0.94Bi0.80Sn0.30Sb in 0.1 M acetate buffer. Figure g) shows the increase in stripping peaks with increasing concentration of the analytes (simultaneously). The full symbols in Figure a,c,e) characterize the linear concentration range, whereas the empty symbols characterize concentrations above and below the linear concentration range.

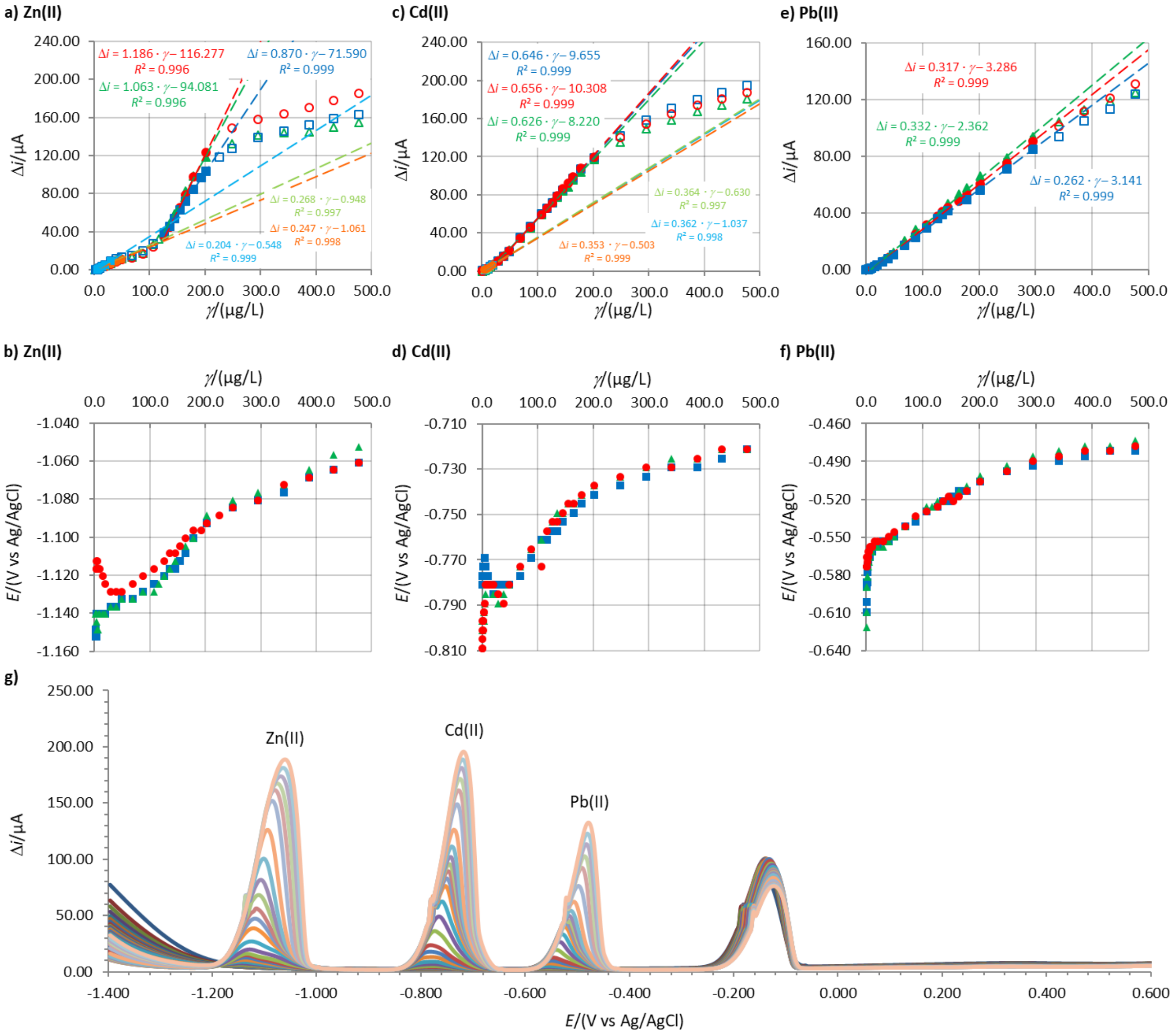

Figure S20: Linear concentration ranges for a) Zn(II), c) Cd(II), and e) Pb(II), and the stripping peak potentials for b) Zn(II), d) Cd(II), and f) Pb(II). The measurements were performed using 0.94Bi0.67Sn0.28Sb in 0.1 M acetate buffer. Figure g) shows the increase in stripping peaks with increasing concentration of the analytes (simultaneously). The full symbols in Figure a,c,e) characterize the linear concentration range, whereas the empty symbols characterize concentrations above and below the linear concentration range.

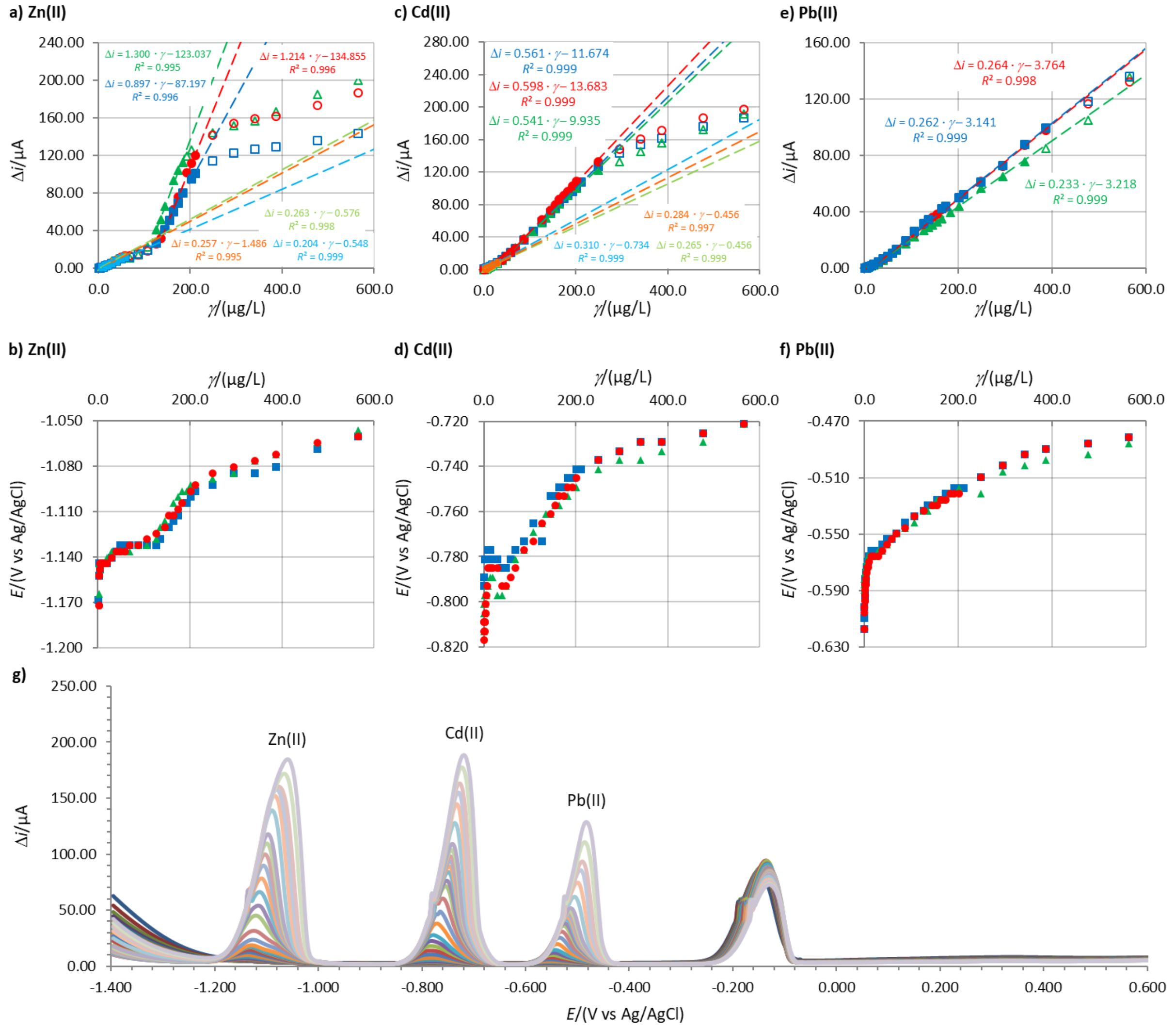

Figure S21: Linear concentration ranges for a) Zn(II), c) Cd(II), and e) Pb(II), and the stripping peak potentials for b) Zn(II), d) Cd(II), and f) Pb(II). The measurements were performed using 1.31Bi0.72Sn0.23Sb in 0.1 M acetate buffer. Figure g) shows the increase in stripping peaks with increasing concentration of the analytes (simultaneously). The full symbols in Figure a,c,e) characterize the linear concentration range, whereas the empty symbols characterize concentrations above and below the linear concentration range.
